# Targeting RCC1 to block the human soft-tissue sarcoma by disrupting nucleo-cytoplasmic trafficking of Skp2

**DOI:** 10.1038/s41419-024-06629-2

**Published:** 2024-04-01

**Authors:** Mingzhi Zhuang, Fengyue Li, Hong Liang, Yongfu Su, Lei Cheng, Bingkai Lin, Jun Zhou, Runzhi Deng, Linying Chen, Peng Lyu, Zhonglei Lu

**Affiliations:** 1 College of Biological Science and Engineering, Fuzhou University, Fuzhou, Fujian 350108 P. R. China; 2 College of Geography and Oceanography, Fuzhou Institute of Oceanography, Minjiang University, Fuzhou, Fujian 350108 P. R. China; 3 Department of Pathology, the First Affiliated Hospital of Fujian Medical University, Fuzhou, Fujian 350005 P. R. China

**Keywords:** Sarcoma, Prognostic markers

## Abstract

Soft-tissue sarcomas (STS) emerges as formidable challenges in clinics due to the complex genetic heterogeneity, high rates of local recurrence and metastasis. Exploring specific targets and biomarkers would benefit the prognosis and treatment of STS. Here, we identified RCC1, a guanine-nucleotide exchange factor for Ran, as an oncogene and a potential intervention target in STS. Bioinformatics analysis indicated that RCC1 is highly expressed and correlated with poor prognosis in STS. Functional studies showed that RCC1 knockdown significantly inhibited the cell cycle transition, proliferation and migration of STS cells in vitro, and the growth of STS xenografts in mice. Mechanistically, we identified Skp2 as a downstream target of RCC1 in STS. Loss of RCC1 substantially diminished Skp2 abundance by compromising its protein stability, resulting in the upregulation of p27^Kip1^ and G1/S transition arrest. Specifically, RCC1 might facilitate the nucleo-cytoplasmic trafficking of Skp2 via direct interaction. As a result, the cytoplasmic retention of Skp2 would further protect it from ubiquitination and degradation. Notably, recovery of Skp2 expression largely reversed the phenotypes induced by RCC1 knockdown in STS cells. Collectively, this study unveils a novel RCC1-Skp2-p27^Kip1^ axis in STS oncogenesis, which holds promise for improving prognosis and treatment of this formidable malignancy.

## Introduction

Soft-tissue sarcoma (STS) has been identified as a heterogeneous mesenchymal tumor, consisting of more than 50 clinical subtypes with variants of genetic background, and histology [1]. Despite incremental advances in diagnosis and management in the past decade, nearly 40% of patients diagnosed with sarcoma ultimately succumb to the disease. A key feature of soft-tissue sarcoma tumor biology is unscheduled S phase entry [2]. Elevated expression of S-phase kinase-associated protein-2 (Skp2), a key regulator of S-phase entry, was found to be associated with a worse prognosis in soft tissue sarcomas [3, 4]. The turnover of Skp2 is reported to be regulated by the anaphase-promoting complex/cyclosome (APC/C) E3 ubiquitinases complex, with CDH1 served as a specific substrate recognition subunit in early G1 phase [5]. Depletion of CDH1 induces upregulation of Skp2 and increased percentage of cells in S phase [6]. On the other hand, Skp2 binds to critical cell cycle proteins, such as p27^Kip1^, and targets them for ubiquitination and degradation [7]. Studies have revealed that the proper function of Skp2-p27^Kip1^ axis depends on its precise subcellular localization and post-transcriptional modification [8]. As such, many oncogenic pathways could promote tumorigenesis by regulating the stability and activity of Skp2. For example, Akt, key component of PI3K/Akt pathway, interacts with and directly phosphorylates Skp2 and triggers its cytoplasmic retention, which in turn promotes the G1/S phase cell cycle transition [7]. Moreover, Akt-mediated phosphorylation also results in a relocation of p27^Kip1^ to the cytosol [8], which further promotes the ubiquitination and degradation of p27^Kip1^. Another report demonstrated that p300-mediated acetylation induces the cytoplasmic localization of Skp2, which stabilizes Skp2 from degradation and further promotes oncogenic signaling in specific settings [8]. Thus, the subcellular translocation process plays important roles in the turnover and the function of Skp2 in the cells. However, the cargo protein that assists the nucleo-cytoplasmic trafficking of Skp2 remains unidentified.

Dysregulation of nucleo-cytoplasmic shuttling would result in abnormal subcellular localization and the aberrant activity of tumor suppressors and oncogenic proteins, thereby contributing to pathological cellular growth [9]. Regulator of chromosome condensation 1 (RCC1), the sole guanine nucleotide exchange factor (GEF) for Ran protein reported, plays a crucial role in coordinating cell mitosis and protein nucleo-cytoplasmic transportation [10, 11]. Mutated RCC1 with persistent activity shows premature condensation of chromosomes [12]. Higher expression of RCC1 is positively associated with cancer progression and poor prognosis [13, 14]. Pavol Cekan and colleagues showed RCC1 overexpression promotes cell cycle progression by increasing the RanGTP-mediated nucleo-cytoplasm transport (NCT) [13]. In contrast, loss of RCC1 blocks the G1/S phase transition without inducing apoptosis in cervical cancer and sensitizes immunotherapy via p27^Kip1^/CDK4 axis [15, 16]. Abundant evidence indicates that the RCC1 acts as an intermediate protein downstream of many oncogenic pathways to promote tumorigenesis. For example, RCC1 was found to be upregulated in E7-expressing epithelial cells and cervical cancer cells [15]. Furthermore, RCC1 knockdown dysregulated the nucleo-cytoplasmic distribution of E2F1, while its overexpression promotes the G1/S phase transition via E2F1-Cdk1 axis [15]. Another study indicated that RCC1 contributes to the trafficking of p27^Kip1^ to the cytoplasm for degradation [14, 17]. Altogether, these studies implicate that RCC1 plays critical roles in cell cycle progression and proliferation of tumor cells. However, the functions of RCC1 in the soft-tissue sarcoma remain unknown.

In the present study, we showed that knockdown of RCC1 induced proliferation inhibition and the arrest of G1/S phase transition in STS cells. We found that loss of RCC1 could downregulate the protein abundance of Skp2 by shortening its half-life in cells. We further employed cell fractionation analysis to show that, RCC1 promoted the cytoplasmic retention of Skp2 in STS cells. Importantly, RCC1 could directly bind to Skp2 and might serve as a cargo protein for the nucleo-cytosol transportation of Skp2 in STS cells. Finally, we validated that suppression of RCC1 could significantly inhibit the growth of soft-tissue sarcoma in vivo, which was largely compromised by introduction of nondegradable form of Skp2. These data provide new insights into the mechanisms of nucleo-cytoplasmic transportation of Skp2 protein, and suggest that RCC1 might play an oncogenic role in STS through inducing cytoplasmic retention and stabilization of Skp2 to promote cell cycle transition and proliferation of STS cells.

## Methods & material

### Bioinformatics analysis and verification

RNA sequence profiles of 412 soft-tissue sarcoma patients were downloaded from the Cancer Genome Atlas (TCGA) databases (https://cancergenome.nih.gov/) by using TCGABiolinks. All statistical analyses were performed using R Studio. The survival maps were generated by Gene Expression Profiling Interactive Analysis2 (GEPIA2, http://gepia2.cancer-pku.cn/#index). Median was selected as a threshold for separating high-expression and low-expression cohorts. Expression levels were further divided into high and low levels using the median expression level as the cut-off point for Kaplan-Meier survival analysis. Results were compared by log-rank test. Patients were stratified into two groups based on the expression levels of RCC1 as described above. The differential expression genes between RCC1-low group and RCC1-high group were obtained with fold change > 2, ranking in the top 10% and *p*-value < 0.05. Gene Set Enrichment Analysis (GSEA) algorithm was used to analyze the genes/pathways associated with RCC1 expression. Co-expression gene networks for cell-cycle, apoptosis, chemokine signaling, and sphingolipid pathways were constructed from the normalized DEGs by with cluster Profiler [18].

### Cell culture and reagents

Cell lines were cultured in DMEM (HEK 293 T) or DMEM F12 (SW872, HTC75) supplemented with 10% fetal bovine serum (FBS), and 100 U/mL Penicillin/ Streptomycin (P/S). All cells were grown at 37 °C in a humidified incubator at 5% CO_2_ and regularly tested for the absence of mycoplasma. Chemical reagents, such as DMSO, NaCl, SDS, and Tris base for buffer preparation were obtained from Sangon Biotech (Shanghai). The FBS, cell culture media were purchased from Hyclone. Antibodies against cyclin A2 and MCM3 were purchased form Santa Cruz Biotechnology. Antibodies against his-tag, Ki67, tubulin and flag-tag were purchased from Abcam. Antibodies against Skp2, myc-tag, p27^Kip1^, rabbit IgG, and mouse IgG in western blot experiments were obtained from Cell Signaling Technology (Beverly, MA). Antibodies against Skp2, myc-tag, used in Co-IP experiment were purchased from Proteintech (Chicago, IL, USA). Anti-BrdU antibody for immunofluorescence staining was obtained from Sigma. HRP-conjugated secondary antibodies against rabbit IgG and mouse IgG were purchased from Abcam.

### Plasmid construction

Flag-CDH1 and Flag-RCC1 were generated by cloning the corresponding cDNAs into p3 × Flag-CMV vector. Briefly, Flag-CDH1 was subcloned with HindIII and EcoRI sites and Flag-RCC1 was subcloned with BglII and SalI sites. Skp2^WT^ cDNA was subcloned into Lenti-myc-CMV vector with AgeI and SalI sites. Site-directed mutagenesis to generate Skp2 mutants (Skp2^Δ45^,Skp2^AA^) was performed using the QuikChange XL Site-Directed Mutagenesis Kit (Stratagene) according to the manufacturer’s instructions.

### shRNAs and transfection

We employed lentivirus infection mediated short-hairpin RNA transduction to generate stable knockdown cells for all gene knockdown experiments throughout the studies. We seeded HEK 293 T in 6 cm dishes at 1 × 10^6^ cells concentration and cultured it in DMEM supplemented with medium for 24 hours. The lentivirus system was applied to transduce the short-hairpin RNA targeted to RCC1 (shRCC1) to knockdown the endogenous expression of RCC1. A scramble sequence not known to target any human genes served as negative control. After 6 hours of transfection, the culture medium was replaced with DMEM medium plus 10% fetal bovine serum. The lentivirus particles were collected 48 hours after transfection and infected the SW872 and HTC75 cell lines, respectively. The stable knockdown cells were established with a puromycin selection (2 μg/mL).

### Cell proliferation analysis

Cell proliferation was recorded with Real-time Cellular Analysis (RTCA) assay. Briefly, cells were plated in E-plated of RTCA analyzer (5 × 10^3^ cells/well) in triplicates, and the growth curves were recorded for 120 hours. On the other hand, the continuous growth images of indicated cells were photographed by ZenCell imaging system at 2-hour-interval. The proliferation curve is generated by GraphPad Prism 9. All assays were performed in triplicates and presented as mean ± SD from three independent experiments.

### BrdU incorporation assay

Cells were plated in 12-well plate for 12 hours, and then treated with 50 μM BrdU (Sigma, B5002) for 2 hours at 37 °C. After fixation by prechilled methanol for 10 min, cells were further incubated in 2 M HCl for 45 min at 37 °C. Blocking was performed with PBS-BSA for 1 hour. Immunostaining for BrdU was further performed with Anti-BrdU (Millipore, ZNA61) at 1:100 dilutions, and subjected to Alexa-594conjugated secondary antibodies (abcam, ab150116) to detect BrdU positive cells. Images were acquired at room temperature using Nikon Ti-S microscope system (Nikon), Nikon DigiSight Digital Camera Head and Nikon NSI-Elements Version 3.10 software. The percentage of BrdU incorporation of indicated cells was determined by ImageJ software using DAPI immunofluorescence as a counterstain.

### FACS cell cycle analysis by BrdU/PI labling

Indicated cells were seeded in 6 cm plate at the density of 1 × 10^6^ cells/well, and serum-staved for 24 hours. Subsequently, cells were labeled with 50 μM BrdU for 2.5 hours. Then, the cells were washed twice with prechilled PBS. Cell pellets were obtained from centrifuge (120 × g for 5 min, 4 °C) then fixed in 1 mL of ice-cold 70% ethanol at 4 °C. After 12 hours, cells were centrifuged and resuspended in 1 mL of 2 M HCl, 0.5% Triton X-100 for DNA denaturation. The reaction was terminated with 1 mL of 0.1 M Sodium Borate (pH = 9.0) for 2 min and centrifuged to remove the neutralized supernatant. The cell pellets were then resuspended in 200 μL /PBS (0.2% BSA, 0.5% Tween 20) and incubated for 5 min, followed by incubation with anti-BrdU antibody (BU20A, eBioscience^TM^, 10 μL per test in 100 μL PBS with 0.2% BSA) for 60 min and counter-staining with propidium iodide (PI) (concentration : 10 μg/mL). Subsequently, the labeled samples were subjected to cell cycle analysis using flow cytometry (BD FACS Verse), and the data was processed with FlowJo software (version 10.8.1).

### Cell migration and invasion assays

Transwell migration assay was applied to identify the cell motility of indicated cells. After 36 hours of serum starvation, the cells were synchronized in G0/G1 phase, then 2 × 10^5^ cells were plated on a polycarbonate membrane with 8.0 μm pore size (Corning, 3422) in the upper chamber of a 24-well plate that contains 200 µL serum-free medium. The bottom chamber contained growth medium with 10% FBS. Then cells were incubated at 37 °C for 24 hours. The migrated cells at bottom of the upper chamber insert was fixed with 100% methanol and stained with crystal violet. The cells on the inner membrane of the upper chamber were gently removed. The images of invaded cells were captured and analyzed. For the invasion assay, the upper chamber was coated with Matrix gel (Corning, 356231) to mimic the extracellular matrix (ECM) of the tumor microenvironment, and cells were then seeded onto the gel after it had coagulated.

### Quantitative real-time PCR

Total RNA was extracted using TRIzol (Invitrogen) according to the manufacturer’s instructions. The HiScript II 1st Strand cDNA Synthesis Kit (Vazyme) was used to reverse transcribe 2 μg RNA into cDNA. Quantitative real-time polymerase chain reaction (qRT-PCR) was performed with a real-time fluorescence quantitative PCR system (Bio-Rad, CFX96 Touch) using a SYBR Premix ExTaq kit (Takara, Dalian, China) under the following conditions: 50 °C for 2 min, 95 °C for 2 min followed by 40 cycles of 95 °C for 15 s and 60 °C for 30 s, with a final cycle consisting of 95 °C for 1 min, 60 °C for 30 s and 95 °C for 15 s. The oligonucleotide sequences of qRT-PCR primers are listed in Supplementary Table 1.

### Western Blotting assay

Indicated cells were seeded in 10-cm plates with 50–60% confluence and incubated for 24 hours. For the protein stability assay, related cells were treated with cycloheximide (CHX) for 0, 2, 4, and 8 hours. Then the cells were harvested, pelleted by centrifugation, and then resuspended in RIPA buffer containing protease inhibitor and 1% Phenylmethanesulfonyl Fluoride (PMSF), and the protein concentration of the cell extract was calculated by the BCA protein estimation assay (Thermo Scientific). Equal amount of protein was loaded and separated by 10% SDS-PAGE, and transferred onto PVDF membranes (Millipore). The protein bands were probed with antibodies with proper dilution and incubated at 4 °C overnight, followed by incubation with peroxidase-conjugated secondary antibodies (1:5,000) for 1 h at room temperature. The bands were visualized by enhanced chemiluminescence. Tubulin was used as loading control.

For the sub-cellular fractionation assay, proteins of cytoplasmic and nuclear proportion of shRCC1 or shScr cells were separated using “Nuclear and Cytoplasmic Protein Extraction kit” (Beyotime Biotechnology). Protein samples of nuclear and cytoplasmic proportions were further subjected to western blotting analysis as described. Lamin B1 was used as loading control for nuclear samples. Tubulin was used as loading control for cytoplasmic samples.

### Co-immunoprecipitation

For the exogenous co-immunoprecipitation, HKE 293 T cells were transfected with the vectors harboring indicated genes. Forty-eight hours after transfection, the proteins of transfected were extracted using immunoprecipitation (IP) buffer (250 mM NaCl, 50 mM Tris, 0.5 mM EDTA, 0.5% NP-40) supplemented with 100 mM phenylmethylsulfonyl fluoride (PMSF). Supernatant of treated groups were collected by centrifugation at 13,000 × *g* for 20 min. Then, 500 µg protein from each sample was incubated with 1 µL of the indicated antibody and 20 μL of protein A-agarose (Santa Cruz Biotechnology) overnight at 4 °C with rotation. Whole cell lysates were used as loading control. The protein A-agarose beads were washed with IP buffer for three times and collected by centrifugation. Eluted protein samples were subsequently subjected to SDS-PAGE and western blotting analysis. RCC1 was detected by monoclonal anti-flag antibody (M2) (1:1,000; Sigma). Skp2 protein were detected with polyclonal anti-myc antibody (1:5,000; Santa Cruz Biotechnology).

For the endogenous co-immunoprecipitation, total proteins from shRCC1 or shScr cells were extracted, processed, and analyzed following the same protocol mentioned above.

### In vivo ubiquitination assay

For in vivo ubiquitination assay, the coding sequence of Myc-tagged Skp2 and 6xhistidine (6 × His)-tagged ubiquitin were cloned into the pLKO.1 vector, respectively. HEK 293 T cells were subsequently co-transfected with these constructs along with shRCC1 for 36 hours, followed by incubation with 20 μM MG132 for 6 hours to prevent the degradation of ubiquitinated proteins. The harvested cells were lysed using a denaturing buffer(6 M guanidine-HCl, 0.1 M Na_2_HPO_4_/NaH_2_PO_4_) and 10 mM imidazole (pH 8.0) to ensure sufficient solubilization. The lysates were then incubated with nickel beads for 3 hours for enrichment of His-tagged proteins. Following extensive washing, the ubiquitinated proteins were eluted from nickel beads and subjected to immunoblotting analysis.

### Immunofluorescence microscopy

Immunofluorescence was performed as previously described [19]. Briefly, shRCC1 or shScr cells were grown on glass cover-slips for 24 hours. Then the cells were fixed with 4% formaldehyde, permeabilized with 1:1 methanol–acetone. The fixed cells were incubated with 5% BSA at room temperature for 1 hours and incubated with anti-BrdU or anti-Skp2 antibodies in a humidified chamber overnight at 4 °C, followed by incubation with HRP-tagged anti-rabbit IgG antibody.

Images were obtained using the confocal microscope (Nikon A1). Quantitative analysis of microscope image was performed using Image J software. For each group, we circled and quantified at least 60 cells from at least 3 independent areas, by measuring the mean fluorescence intensity of nucleus area and cytoplasm area separately.

### Mice xenograft tumor model establishment and treatment

All the animal experimental procesures in this study were approved by Fuzhou University Animal Care Committee, conforming to accepted standards of humane animal care (Approval protocol ID: 2019-SG-022). Mice were randomly divided into two groups—the Scramble group and the shRCC1 group—with 8 mice per group, and a total of 8 × 10^6^ indicated cells (shScr, shRCC1, Skp2^AA^ and shRCC1+Skp2^AA^) cells were suspended in 100 μL of prechilled PBS and transplanted by subcutaneous injection (s. c.) into the flanks of 4-week-old male nude mice. One week after implantation, xenograft size was continuously measured twice a week by caliper and calculated using the following equation: Volume = 0.5 × length × width^2^. The mice were sacrificed in day 28 after implantation. The harvested xenografts were measured and weighed, and then were stored in −80 °C or fixed in10% formalin.

### Immunohistochemistry (IHC) of formalin fixed, paraffin embedded (FFPE) tissues

Sections (4 μm) obtained from mice FFPE xenograft tissues were subjected to IHC analysis to evaluate the expression of Skp2, Ki67, RCC1, and p27^Kip1^. Following deparaffinization and rehydration, antigen retrieval was performed with citrate buffer (pH = 6.0). Then, the sections were treated with peroxidase blocking solution (3% H_2_O_2_) for 10 minutes at room temperature. IHC was performed using indicated antibody, with the sections being incubated with the antibody at recommended dilutions for 2 hours at room temperature. IHC staining was achieved using DAB: ImmPACT DAB Peroxidase (HRP) Substrate (Thermo Fisher) for about 4 minutes at room temperature, followed by counterstaining with hematoxylin for 1 min at room temperature. Sections were dehydrated, mounted and subjected to image acquisition using an optical microscope.

### Statistical analysis

All data analyses were performed using GraphPad (version 8.0). The data are presented as mean ± SD from at least three independent experiments. Two-way unpaired Student’s t-test or one-way ANOVA followed by the Bonferroni test was used for comparisons between two groups or multiple groups, respectively, except specifically noted.

## Results

### RCC1 is an oncogene in STS and is correlated with poor prognosis of STS patients

**Fig. 1:**
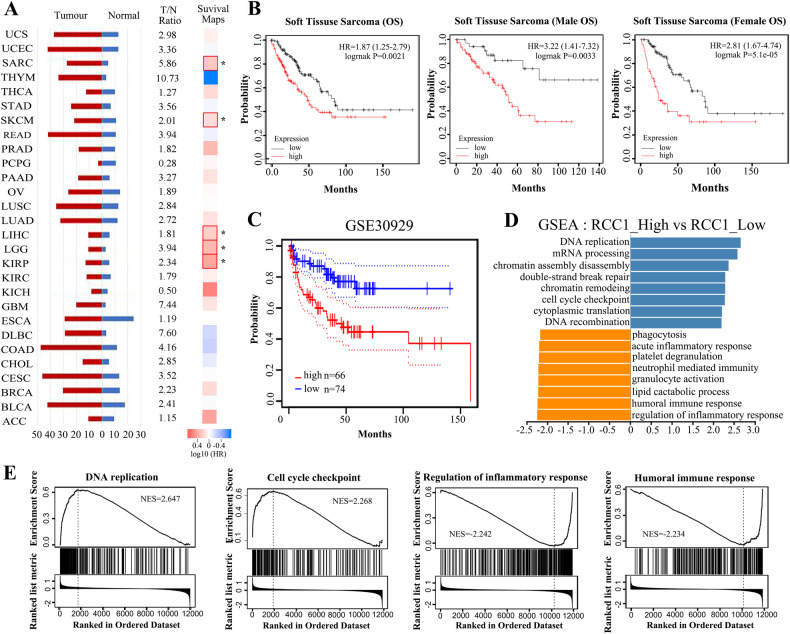
The upregulation of RCC1 is correlated with poor prognosis of STS patients. **A** The expression level of RCC1 across various cancer types in TCGA database,, along with the Tumor-to-Normal expression ratio. Survival map of hazard ratio (HR) shows the prognostic impacts of RCC1 on multiple cancer type. The red and blue blocks represent higher and lower risks, respectively. The bounding boxes depicted the significant (*p* < 0.05) unfavorable and favorable results, respectively. **B** The prognostic analysis between high and low expression of RCC1 in TCGA database. **C** Kaplan-Meier analysis for RCC1 expression in GSE 30929 patient cohort. **D**, **E** Gene set enrichment analysis (GSEA) showed that the samples with high RCC1 expression enriched in DNA replication (NES = 2.647), cell cycle checkpoint (NES = 2.268). The most downregulated gene sets with high RCC1 expression was clustered in regulation of inflammatory response (NES = −2.242), humoral immune response (NES = −2.234).

The expression level of RCC1 was reported to be elevated in multiple types of cancers [20]. Indeed, through a joint bioinformatics analysis with TCGA and GTEx database, we found that RCC1 is more abundantly expressed in tumor tissues compared to adjacent normal tissues in thymoma (THYM, T/N Ratio = 10.73), glioblastoma (GBM, T/N Ratio = 7.44), large B-cell Lymphoma (DLBC, T/N Ratio = 7.60), and sarcoma (SARC, also referred as STS, T/N Ratio = 5.86). Among the top 4 cancer types with highest T/N ratio of RCC1 expression (THYM, GBM, DLBC, SARC), only SARC is identified with a significant reduced survival in patients (Fig. 1A). We then analyzed the correlation between RCC1 expression and the survival of STS patients by using the TCGA-SARC database, and found that higher RCC1 expression is indeed correlated with shorter survival time in STS patients, without gender difference (Fig. 1B). To further evaluate the prognostic potential of RCC1 in clinic STS patients, the raw microarray data and clinical information of STS patient cohort was downloaded from the NCBI GEO database (accession No. GSE30929). A total of 140 primary sarcoma samples were stratified into the RCC1-high group and RCC1-low group based on the mean value of RCC1 abundance [21]. The results of Kaplan-Meier survival analysis illustrated that higher RCC1 expression was indeed correlated with shorter overall survival in this patient cohort (Fig. 1C). Furthermore, we identified the distinct transcriptomic profiles between the RCC1-high and RCC1-low groups. This identified 24 out of the 163 significantly differentially expressed genes represented in the RNA-seq data set to be cancer associated. The significant different genes with satisfying threshold value (*p*-value < 0.01, |logFC | ≥ 1) are conspicuously denoted and visualized as red and blue dots, respectively (Fig. S1A). Subsequently, we conducted Gene Set Enrichment Analysis (GSEA) on the transcriptomic profiles of the high and low groups, utilizing gene sets from the Molecular Signatures Database (MSigDB 7.0) [22]. Based on the results, the top 10 enriched gene sets, positively or negatively correlated with RCC1 expression, were shown in Fig. 1D (*p* < 0.05). The enriched gene sets correlated with the RCC1 expression variation comprise several biological processes: DNA replication (NES = 2.647), chromatin assembly or disassembly (NES = 2.356), cell cycle checkpoint (NES = 2.268), regulation of inflammatory response (NES = −2.242), and humoral immune response (NES = −2.234), (Fig. 1E and S1B, details listed in Supplementary Table 2). These results suggest that RCC1 plays oncogenic roles in STS, and indicates a poor prognosis in STS patients. RCC1 might be involved in the processes of cell cycle regulation, cell proliferation and inflammatory response to promote the oncogenesis of STS.

### Knockdown of RCC1 impairs the cell growth and motility of STS cells

**Fig. 2:**
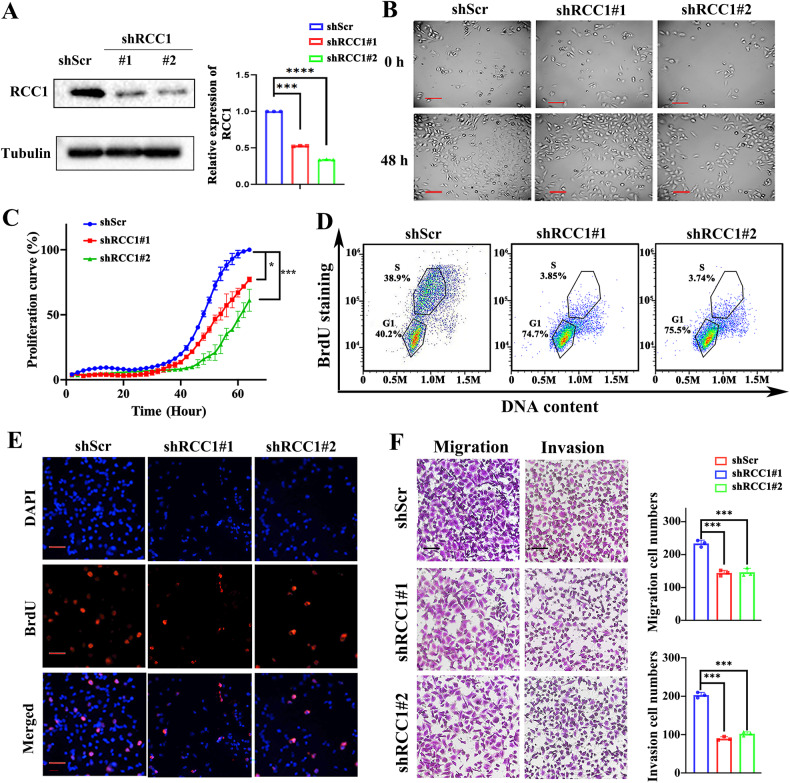
Knockdown of RCC1 represses the cell growth and motility of STS cells. **A** Western blot analysis of RCC1 stable knockdown soft tissue sarcoma cell line SW872 by lentivirus mediated stable transduction (shScr: sh-scramble). **B** Endpoint bright field images of RCC1 knockdown SW872 cell lines by using live cell imaging system ZenCell owl. **C** Real-time cellular analysis (RTCA) of RCC1 knockdown SW872 cell lines. **D** FACS analyses using BrdU and PI staining for shScr and RCC1-KD SW872 cells. The cells belong to different cell cycle sub-phase was determined within the circle, with the percentage shown close to it. **E** Immunofluorescence images of indicated cells stained by pulse-incorporation of BrdU and immunostaining using anti-BrdU antibody (red). DAPI was used to counterstain the cells nuclei (blue). **F** Knockdown of RCC1 decreased migration and invasion of SW872 cells. The cells in five randomly selected fields were counted and statistically analyzed. The number of migration/invasion cells per field was fewer in shRCC1 cells compared with shScr cells. Data is expressed as mean ± SD (*n* = 3). Significance (*p < 0.05, ***p < 0.001). Scale bars: 100 μm.

We have shown that RCC1 is positively correlated with the poor prognosis of clinical STS patients. We next employed loss-of-function studies to investigate whether knockdown of RCC1 would compromise the cell cycle progression, proliferation, and migration of STS cells in vitro. To this end, we efficiently knockdown the expression of RCC1 in two STS cell lines, SW872 and HTC75, by lentivirus-mediated transfection of two shRNAs targeting RCC1 (Figs. 2A and S2A). By using the ZenCell living cell monitor system, we found that downregulation of RCC1 significantly compromised the growth of both SW872 and HTC-75 cells 48 hours after passage (Figs. 2B and S2B). The Real-time cell analyzer (RTCA) was further employed to record the growth curves of SW872 and HTC75 cells, and showed that knockdown of RCC1 continuously inhibits the proliferation of STS cells throughout the monitoring period (Figs. 2C and S2C). To determine if this proliferation inhibition was due to the cell cycle blockage, we applied the 5-bromo-2′-deoxyuridine (BrdU) incorporation FACS assay to evaluate the G1/S phase transition of STS cells following RCC1 knockdown. As expected, knockdown of RCC1 dramatically compromised the BrdU incorporation in STS cells, while the proportion of G0/G1 phase increased from 40.2% to 74.7% (Figs. 2D and S2D). We also observed a significant decrease in the proportion of BrdU-positive cells by RCC1 silencing, using immunofluorescence assay of STS cells following BrdU pulse-labeling (Figs. 2E and S2E). Besides growth inhibition, we also found that RCC1 knockdown significantly impaired the migration and invasion potential in both SW872 and HTC75 cells, compared to the shScr group (Figs. 2F and S2F). Together, these results suggest that RCC1 might function as an oncogene in soft-tissue-sarcoma cells by promoting the cell proliferation, migration and invasion.

### Knockdown of RCC1 compromises the protein stability of Skp2 in STS cells, leading to the accumulation of p27^Kip1^

**Fig. 3:**
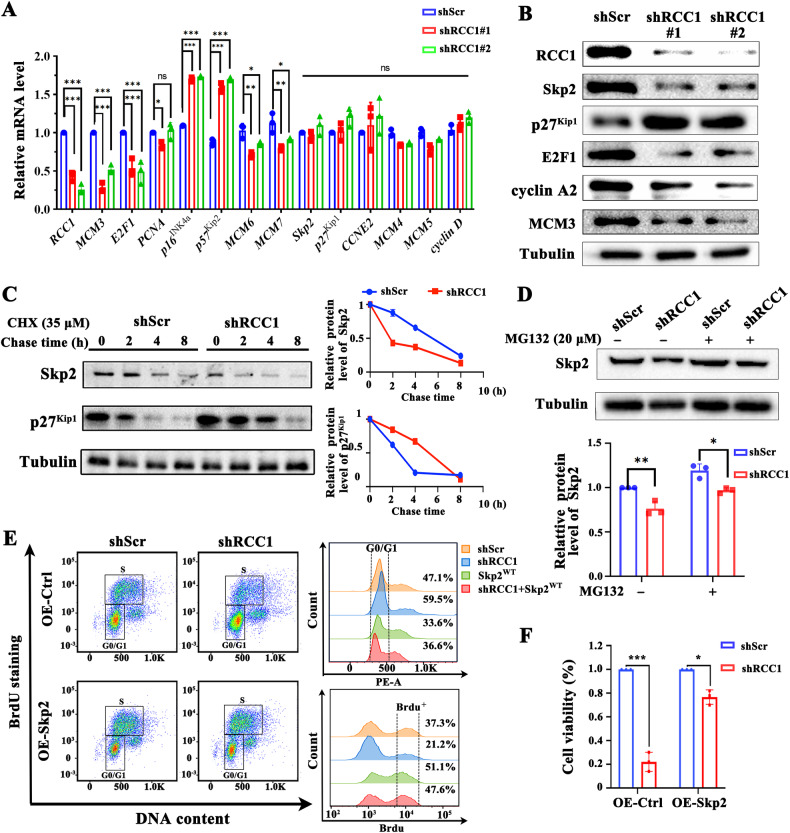
Knockdown of RCC1 reduces the protein stability of Skp2 in STS cells, leading to the accumulation of p27^Kip1^. **A** qRT-PCR for mRNA expression analysis of proliferation marker genes (*Skp2, cyclin D, cyclin R, MCM3, MCM6, MCM7, E2F1, p57, p27*^*Kip1*^*, p16*^*Ink*^*and PCNA*) in RCC1 knockdown (shRCC1#1, shRCC1#2) SW872 cells. **B** Immunoblotting analysis of RCC1, Skp2, p27^Kip1^, E2F1, cyclin A2 and MCM3 in shScr and RCC1 knockdown (shRCC1#1, shRCC1#2) SW872 cells by lentivirus mediated transduction. Tubulin was used as the loading control. **C** Cycloheximide (CHX) assays showed that RCC1 knockdown accelerating the degradation rate of Skp2 in SW872 cells. Indicated cells were treated with cycloheximide (CHX, 35 μM) to inhibit protein synthesis, and harvested at indicated time-points for immunoblotting analysis. Tubulin was used as the loading control. Quantification of Skp2 and p27^Kip1^ abundance normalized to tubulin is shown alongside. **D** Proteasome degradation assay of protein Skp2 in RCC1 knockdown SW872 cells. Cells were treated with or without the proteasome inhibitor MG132 (20 μM) for 6 hours before harvest and western blot analysis. Tubulin was used as loading control. **E** FACS analyses of shScr or shRCC1 SW872 cells transduced with Skp2 overexpression plasmid for 48 hours. The cells belong to different cell cycle sub-phase was determined, with the percentage shown alongside using barchart. **F** The cell proliferation rate of Skp2 overexpression (OE-Skp2) in shScr or shRCC1 SW872 cells was analyzed by CCK8. Data is expressed as mean ± SD (*n* = 3). Significance (*p < 0.05, **p < 0.01, ***p < 0.001).

We have shown that downregulation of RCC1 in STS cells induced the cell cycle arrest in G1/S transition, leading to inhibition of cell growth. To investigate the mechanisms underlying this regulation by RCC1, we performed qRT-PCR to analyze the changes in expression of genes related to G1/S transition. As shown in Fig. 3A, genes involved in G1/S transition, such as MCM3 MCM6, MCM7, and E2F1, downregulated their expression at mRNA level by two hairpins of shRNAs targeting RCC1. In contrast, CDK inhibitors (CKIs) of G1 phase such as p16^INK4a^ and p57 were upregulated in RCC1 knockdown groups. However, the mRNA expression of Skp2, cyclin E and p27^Kip1^ genes were not affected by RCC1 knockdown. As a key regulator in the cell cycle G1/S transition, Skp2 is often regulated at the process of protein turnover [23]. We next analyzed whether RCC1 would regulate the protein expression of these genes. As expected, knockdown of RCC1 dramatically reduced the protein abundance of E2F1, cyclin E, MCM3 and Skp2, while elevated the protein levels of p27^Kip1^ and p16^INK4a^ (Figs. 3B and S3A). In contrast, the protein abundance of β-TrCP, another E3 ubiquitinase functioning in early G1 phase, was not affected upon RCC1 knockdown (Fig. S3A). The results indicate that the loss of RCC1 would induce the protein reduction of Skp2, leading to the accumulation of its ubiquitination target p27^Kip1^. The accumulation of p27^Kip1^, in turn, hinders the G1/S transition in STS cells.

In order to examine whether this reduction of Skp2 protein by RCC1 knockdown is due to protein degradation, the protein stability assay was performed in STS cells. Specifically, STS cells with transduction of shScr or shRCC1 respectively were treated with cycloheximide (CHX) to block protein synthesis, and were harvested at the indicated time-points for immunoblotting analysis of Skp2 and p27^Kip1^. As a result, we found that RCC1 knockdown significantly reduced the turnover half-life of Skp2 protein from 5.4 hours to 1.7 hours, while prolonged the half-life of p27^Kip1^ protein from 2.1 hours to 4.4 hours, indicating that the protein stability of Skp2 is compromised by knockdown of RCC1 (Fig. 3C). We further studied whether this impairment of protein stability of Skp2 is through protein degradation. To this end, we used proteasome inhibitor MG132 to block protein degradation process, and then examined the protein abundance of Skp2 in STS cells with transduction of shScr or shRCC1. As shown in Fig. 3D, while RCC1 knockdown promoted the degradation of Skp2, combined treatment of MG132 largely reversed this effect, suggesting that the protein stability impairment of Skp2 by RCC1 knockdown is indeed through proteasome mediated protein degradation process.

To further investigate whether the induced degradation of Skp2 is functionally related to the cellular phenotypes induced by RCC1 knockdown in SW872 cells, we stably overexpressed Skp2 in the established shScr- and shRCC1-SW872 cell lines. Subsequently, we employed FACS analysis and CCK8 cell viability assay to examine whether restoration of Skp2 protein would recover the cell cycle G1/S phase transition and cell growth impaired by loss of RCC1.

Analysis revealed that the shRCC1 group exhibited a 12.4% increase in the proportion of G1 phase cells compared to the shScr group. In contrast, shRCC1; OE-Skp2 groups displayed a reduction of G1 phase population, to a comparable extent of the shScr group. Furthermore, the percentage of BrdU positive population, which indicates the cells entering into the DNA synthesis phase (S phase), was notably lower in the shRCC1 group (21.2%), compared to 37.3% in the shScr group. However, the proportions of S phase cells were increased to 47.6% in the shRCC1+OE-Skp2 group, suggesting that RCC1 promote the G1/S transition in STS cells through Skp2 (Fig. 3E). As shown in Fig. 3F, while shRCC1 induced significant cell growth inhibition compared to shScr group, combined overexpression of Skp2 led to the recovery of cell growth in shRCC1-SW872 cells.

In addition, we analyzed whether restoration of RCC1 would adversely affect the turnover of the Skp2 protein and the phenotypes in SW872 cells. The results showed that overexpressing RCC1 in RCC1 knockdown cells not only restored the abundance of Skp2 and p27, but also improved the cell viability and the colony formation potential of RCC1 knockdown cells, to a comparable extent of wild-type cells (Fig. S3B–S3D). Furthermore, we observed that the exogenous overexpression of RCC1 restored the G1/S phase transition in RCC1 knockdown cells (Fig. S3E). Together, these data indicate that RCC1 knockdown suppresses the proliferation, migration and invasion of soft-tissue sarcoma cells through compromising the protein stability of Skp2, and that RCC1 and Skp2 might be functionally related in the oncogenesis of soft-tissue sarcoma.

### Knockdown of RCC1 elevates the poly-ubiquitination of Skp2 by promoting its nucleus retention

**Fig. 4:**
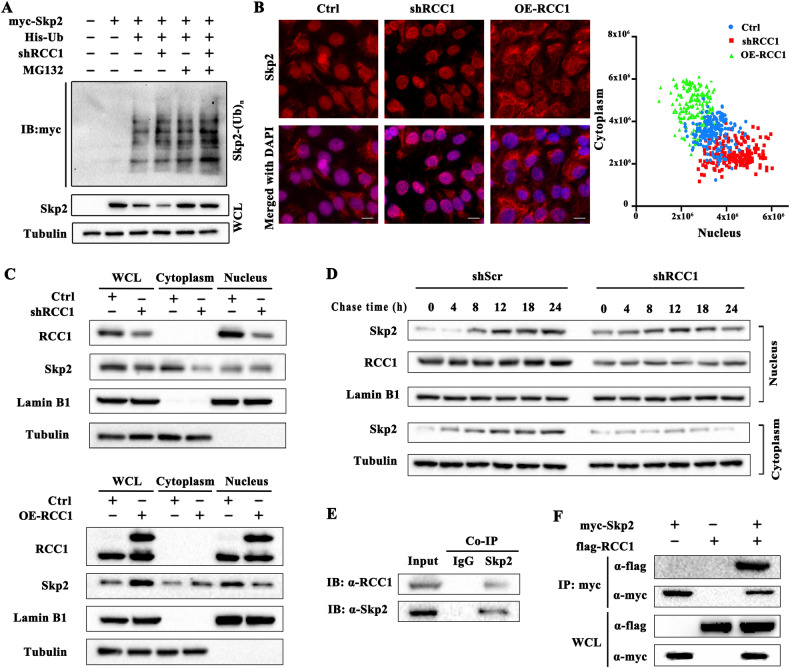
Knockdown of RCC1 elevates the poly-ubiquitination of Skp2 by promoting its nucleus retention. **A** In vivo exogenous ubiquitination assay to detect ubiquitination of Skp2 influenced by RCC1 knockdown on SW872 cell. Each cell group was co-transduced with Myc-Skp2, His-Ubiquitin (His-Ub) and shScr/shRCC1 plasmids as indicated. **B** Immunofluorescence analysis was performed to determine the subcellular localization of Skp2 (red) in SW872 cells with shRCC1 and RCC1 overexpression (OE-RCC1). DAPI was used to counterstain the cells nuclei (blue). The X-Y scatter plot of 200 cells from control (blue dots), shRCC1 (red dots) and OE-RCC1 (green dots) groups based on the quantification of nucleus and cytoplasm fluorescence intensity of Skp2 is shown alongside. Scale bars: 10 μm. Data is expressed as mean ± SD (*n* = 3). Significance (**p < 0.01, ***p < 0.001). **C** Immunoblotting analysis of subcellular localization of Skp2 in shRCC1 or OE-RCC1 SW872 cells. Cells were starved for 48 hours and subsequently released into full culture medium for 12 hours. Lamin B1 was used as nuclear control and tubulin was used as cytoplasmic control. **D** Immunoblotting analysis was conducted to investigate the subcellular localization regulation of Skp2 in RCC1 knockdown SW872 cells throughout the cell cycle process. SW872 cells were transduced with the control plasmid (shScr) or RCC1 knockdown plasmid (shRCC1) were starving for 48 hours and subsequently released into full culture medium for several time period (0, 4, 8, 12, 18, and 24 hours). Cytoplasmic and nuclear extracts were analyzed by immuno-blotting. Lamin B1 was used as nuclear control and tubulin was used as cytoplasmic control. In vivo protein co-immunoprecipitation of endogenous Skp2 and RCC1 in SW872 cells (**E**) and exogenous binding in HEK 293 T cells (**F**).

We further investigated how knockdown of RCC1 compromises the protein stability of Skp2. Anaphase promoting complex/cyclosome (APC/C) complex is a known E3 ligase targeting Skp2 for ubiquitination and destruction through proteasome, and accumulating evidence indicates that it could bind to Skp2 in the nucleus through its substrate recognition subunit CDH1 [24]. In contrast, cytoplasmic localization of Skp2 would inhibit its binding with APC/C-CDH1 complex, and spare it from further ubiquitination and degradation [7, 8]. Firstly, we employed poly-ubiquitination assay to analyze whether knockdown of RCC1 would affect the poly-ubiquitination of Skp2. As shown in Fig. 4A, by transfection of Myc-Skp2 and His-Ub in HEK-293T cells, we established exogenous Skp2 poly-ubiquitination assay with or without MG132. On this basis, we observed that knockdown of RCC1 promoted the poly-ubiquitination of Skp2. The accumulation of poly-ubquitinated Skp2 by RCC1 knockdown was much more evident in the presence of MG132. We next investigated the effect of RCC1 knockdown on the ubiquitination of endogenous Skp2. By immunoprecipitating endogenous Skp2 and probing for ubiquitin, we also observed more accumulated ubiquitinated endogenous Skp2 upon RCC1 knockdown, indicating that RCC1 depletion would downregulate Skp2 by promoting its poly-ubiquitination followed by degradation (Fig. S4A)

Furthermore, we sought to investigate how RCC1 would protect Skp2 from ubiquitination. Based on literatures, APC/C complex with substrate recognition subunit CDH1, is a well known specific E3 ubiquitinase for Skp2. Expression of nondegradable Skp2 mutants, Skp2^Δ45^ (N-terminal (aa1-45) deletion mutants) and Skp2^AA^ (RxxL motif in D-box I was mutated to AxxA), with deficiency in binding with CDH1 would induces premature entry into S phase [6]. Thus, to understand how RCC1 protects Skp2 from ubiquitination, we elucidated the underlying interrelationship between RCC1, Skp2 and CDH1. Firstly, we performed cycloheximide (CHX) assay to compare the protein stability between Skp2^WT^, Skp2^Δ45^ and Skp2^AA^. We found that the abundance of Skp2^WT^ gradually diminished post CHX treatment, indicating a proteasome degradation of Skp2. In contrast, Skp2^Δ45^ and Skp2^AA^, maintain stable in protein abundance during the detection time period (Fig. S4B). Next, we investigate whether RCC1 would protect Skp2 from CDH1 mediated degradation. We showed that although CDH1 promoted the degradation of Skp2^WT^, combined RCC1 overexpression reversed this degradation, to a comparable extent of control group. However, RCC1 overexpression does not further increase the abundance of Skp2^Δ45^ and Skp2^AA^, indicating that the protection of Skp2 from degradation by RCC1 is dependant on CDH1 (Fig. S4C). On the other hand, we analyzed the protein stability between Skp2^WT^, Skp2^Δ45^ and Skp2^AA^, in the context of RCC1 knockdown. As shown in Fig. S4D, although RCC1 knockdown greatly compromised the protein stability of Skp2^WT^, two mutant forms of Skp2, Skp2^Δ45^ and Skp2^AA^, showed enhanced protein stability upon RCC1 depletion. Notably, Skp2^AA^ exhibited a more pronounced resistance to degradation following RCC1 knockdown as compared to Skp2^Δ45^.

Next, we queried whether this elevation in poly-ubiquitination of Skp2 upon RCC1 knockdown would be correlated with its subcellular distribution alteration. We performed immunofluorescence analysis to visualize the subcellular localization of Skp2 protein in SW872 cells with shRCC1 or RCC1-OE transduction. We randomly selected 200 cells from each group (Ctrl, shRCC1 and OE-RCC1 cells) and measured the red fluorescence intensity of nuclear Skp2 and cytoplasmic Skp2. We plotted the 200 cells of each group on a XY plots based on the nuclear and cytoplasmic fluorescence intensity of Skp2. Based on the quantification, we clearly observed a cell population with nuclear Skp2 accumulation in RCC1 knockdown group, while the cell population in OE-RCC1 group exhibited more cytoplasmic Skp2 accumulation (Fig. 4B).

Further, to investigate whether the alteration in the subcellular distribution of Skp2 by OE-RCC1 is due to the effect on the nucleo-cytoplasmic trafficking process, we included exportin 1 (XPO1) as a positive control. XPO1 is a classical nucleo-cytoplasmic shuttling protein, mediating the nucleus export of various RNA species and proteins [25]. Importantly, RCC1 is reported as the exclusive regulatory protein mediating the nucleo-cytoplasmic trafficking of XPO1 [26]. We conducted subcellular fractionation and immunofluorescence to examine the nucleo-cytoplasmic trafficking of Skp2, side-by-side with XPO1, following the expression of RCC1. As shown in Fig. S4E and S4F, we found that in cells overexpressing RCC1, XPO1 is predominantly located in the cytoplasm, consistent with the increased cytoplasmic distribution trend of Skp2. These results demonstrate that RCC1 mediates the nucleo-cytoplasmic trafficking of Skp2.

We further performed subcellular fractionation assay in SW872 cells with RCC1 overexpression or RCC1 knockdown, and quantified the nuclear and cytoplasmic Skp2 abundance respectively. Cells of indicated groups (Ctrl, shRCC1, and OE-RCC1) were initially synchronized to the G0/G1 phase by serum starvation, released into serum for 12 hours, a time point close to G1/S transition, and harvested for western blot analysis. The results showed that, at 12 h post serum release, RCC1 knockdown induced a mild nucleus accumulation of Skp2 as compared to Ctrl group, while the cytoplasmic fraction showed dramatic reduction of Skp2 abundance. As a result, we observed a slight reduction of total Skp2 abundance in whole cell lysates (WCL) of shRCC1 group compared to Ctrl group, indicating that RCC1 knockdown would induced nucleus accumulation of Skp2 in early time points followed by degradation at later time points (Fig. 4C). On the other hand, RCC1 overexpression groups exhibited a significant upregulation and cytoplasmic distribution of Skp2, further supporting the role of RCC1 in modulating Skp2 subcellular localization.

This phenomenon is consistent with the results shown in Fig. 4D, in 0–8 h time period, there are slightly more nuclear Skp2 accumulation in RCC1 knockdown group compared to shScr group. However, in 12–24 h time period, nuclear Skp2 in RCC1 knockdown cells become less than shScr group. In contrast, cytoplasmic Skp2 maintained at lower lever in RCC1 knockdown group at all timepoints. The results suggest that RCC1 depletion might inhibit and delay the nucleo-cytoplasmic shuttling of Skp2 in early G1 phase to ensure sufficient ubiquitination of Skp2 by APC/C-CDH1 [7, 8], followed by more efficient proteasomal degradation of Skp2 in cytoplasmic portion of STS cells.

RCC1 is well known for facilitating the nucleo-cytoplasmic shuttling of target proteins by promoting the formation of the trimeric exportin–receptor–RanGTP complex [27]. Whether it participates in the nucleus exportation of Skp2 has not been reported yet. To further elucidate the potential role of RCC1 in promoting the cytoplasmic distribution of Skp2, we utilized protein co-immunoprecipitation (co-IP) assay to examine whether RCC1 would directly bind to Skp2 protein. As expected, we showed that, at endogenous level, RCC1 could bind to Skp2 in SW872 cells (Fig. 4E). Further, we established an exogenous system by transfecting HEK-293T cells with Flag-RCC1 and Myc-Skp2, and observed a more robust interaction between RCC1 and Skp2 (Fig. 4F). Finally, we investigated whether RCC1 would protect Skp2 from CDH1 mediated degradation through direct binding with Skp2. The literature indicates that, Skp2^Δ45^ and Skp2^AA^ are deficient in CDH1 recognition and thus resistant to CDH1 mediated degradation [6]. We also showed that Skp2^Δ45^ and Skp2^AA^ exhibit resistance to CDH1 mediated degradation (Fig. S4B and S4C). On this basis, we performed co-IP assays to assess the interaction between RCC1 and Skp2^Δ45^ and Skp2^AA^. We showed that RCC1 could directly bind to Skp2^WT^ and Skp2^AA^, while lost the ability to bind to Skp2^Δ45^ mutant (Fig. S4G). These results suggest that RCC1 might compete with CDH1 to bind to the D-box domain of Skp2, thus interfere with the CDH1 mediated degradation of Skp2.

### Knockdown of RCC1 inhibits the growth of STS xenograft in mice by facilitating the degradation of Skp2

After validating the non-degradable characteristic of Skp2^AA^ (Fig. S4B–S4D), we further conducted a series of functional experiments in vitro to study whether expression of non-degradable Skp2^AA^ would resume the cancer progression of RCC1 knockdown cells. Our findings indicated that RCC1 knockdown suppresses STS cell proliferation in vitro through the enhanced degradation of Skp2. Following the introduction of nondegradable Skp2^AA^, we observed a significant rescue effect in cell viability, colony formation ability, cell proliferation rates and G1/S transition in RCC1 knockdown cells (Fig. S5A–D). These results demonstrate that expression of non-degradable form of Skp2 indeed resumes the cancer progression of RCC1 knockdown cells to a comparable extent of wild type cells.

**Fig. 5:**
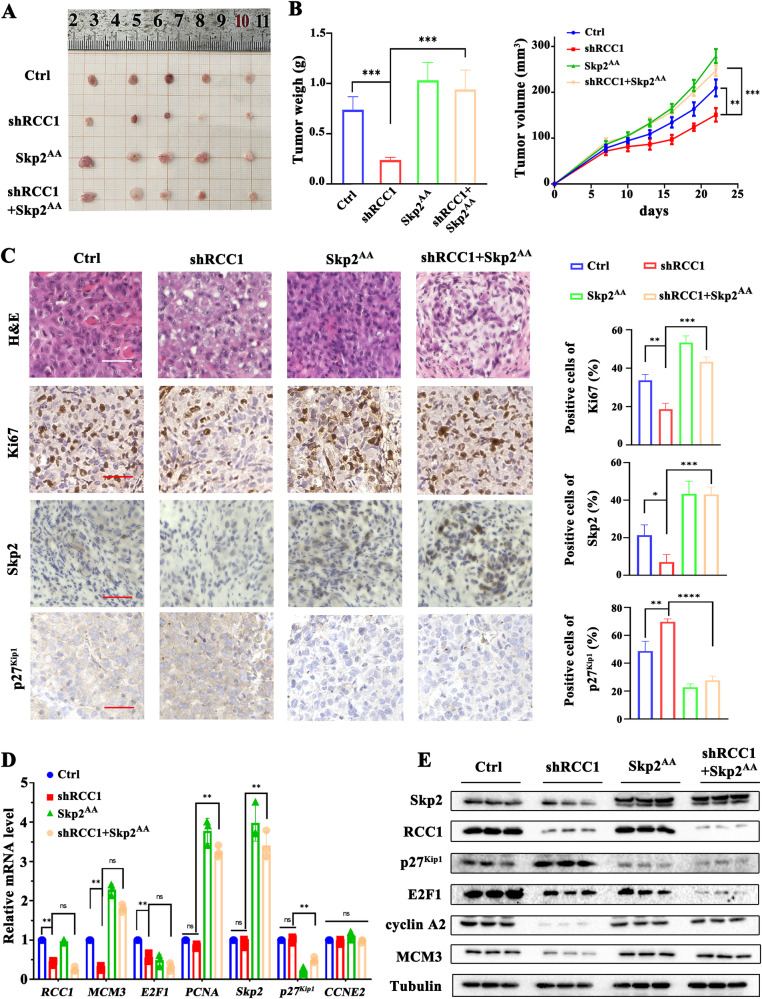
Knockdown of RCC1 inhibits the growth of STS xenograft in mice. **A** Photograph of xenograft tumors from shScr, shRCC1, Skp2^AA^ or shRCC1+Skp2^AA^ groups harvested at the endpoint. **B** Growth curves of inoculated xenograft tumors and average tumor weight at the endpoint from shScr, shRCC1, Skp2^AA^ or shRCC1+Skp2^AA^ groups. Data are expressed as mean ± SD (*n* = 5, **p < 0.01, ***p < 0.001). **C** Representative H&E images and immunohistochemistry (IHC) of Ki67, Skp2 and p27^Kip1^ of xenograft tumor sections from shScr, shRCC1, Skp2^AA^ or shRCC1+Skp2^AA^ groups. Percentage of IHC positive cells are quantified alongside. Scale bars: 50 μm. **D**, **E** Relative mRNA and protein abundance of *Skp2* and marker genes (MCM3, E2F1, PCNA, Skp2, p27^Kip1^, CCNE2) of related pathway in harvested xenograft tumor samples (shScr, shRCC1, Skp2^AA^ or shRCC1+Skp2^AA^). Data are expressed as mean ± SD (*n* = 3, *p < 0.05, **p < 0.01, ***p < 0.001).

Next, we sought to investigate whether expression of non-degradable Skp2^AA^, would resume the tumor volume, as well as cancer phenotypes, in established xenograft STS tumor model in nude mice. We established STS xenograft models by inoculating SW872 cells stably transduced with shScr, shRCC1, Skp2^AA^ or shRCC1+Skp2^AA^, respectively. The tumor burdens of xenografts were monitored and recorded every 72 hours. Four weeks after implantation, the mice were sacrificed with the xenografts being carefully harvested, weighed and photographed. We observed that the average xenograft volume of the shRCC1 group was markedly reduced compared to shScr group. However, combined overexpression of non-degradable Skp2^AA^ with RCC1 knockdown significantly resumed the tumor growth in shRCC1+Skp2^AA^ group (Fig. 5A, B). The H&E examination of harvested xenografts from above mentioned groups further revealed that while shRCC1 induced significant H&E apoptosis phenotype, combined overexpression of nondegradable Skp2^AA^ with shRCC1, significantly resumed H&E cancerous phenotype, represented as more pronounced pleomorphism and increased mitotic activity in shRCC1+Skp2^AA^ group. Moreover, IHC analysis revealed a marked reduction of proliferation markers (Ki67) and Skp2 abundance in the shRCC1 group compared to shScr group. However, combined expression of nondegradable Skp2^AA^ with shRCC1 (shRCC1+Skp2^AA^ group) significantly restored the Skp2 abundance and Ki67 positive rate to a comparable extent of shScr group (Figs. 5C and S5E). Interestingly, we observed elevated apoptosis in shRCC1 groups by TUNEL assay, which is also reversed with combined expression of Skp2^AA^ (Fig. S5F). Consistently, relative RNA quantification and western blot analysis with ex vivo xenograft samples confirmed that combined overexpression of Skp2^AA^ significantly restored the expression of marker proteins related to cell proliferation and G1/S phase transition, including E2F1, MCM3, and cyclin A2, in RCC1 knockdown cells (Fig. 5D, E). Together, these functional studies with nondegradable Skp2^AA^ demonstrate that knockdown of RCC1 could suppress the growth of STS tumor in vivo by downregulating the Skp2 abundance to block the cell proliferation.

## Discussion

**Fig. 6:**
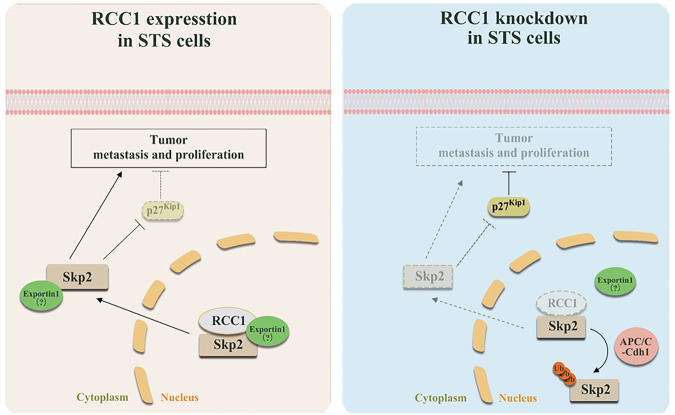
Proposed model for knockdown of RCC1 induced Skp2 nuclear retention and degradation, subsequently influences its oncogenic function. In this model, the present of RCC1 maintained the nuclear-cytoplasmic process of Skp2 in soft-tissue sarcoma. Decreased RCC1 sequesters Skp2 within nuclear, which results in the subsequent ubiquitination and degradation of Skp2. Lack of cytoplasmic Skp2 restored the expression of p27^Kip1^, and then retarded the proliferation, migration of soft-tissue sarcoma.

The genetic heterogeneity, local recurrence, and high metastatic rate, present obstacles to effective treatment for advanced or metastatic STS [28]. In this study, we demonstrated that elevated RCC1 expression is associated with an unfavorable prognosis in patients with STS. Knockdown of RCC1 significantly repressed the proliferation and migration of STS cell lines in vitro, and the growth of STS xenograft tumors in vivo. We further identified that RCC1 depletion downregulates the abundance of Skp2 protein by compromising its protein stability in STS cells. Mechanistically, we showed that RCC1 knockdown induced nuclear retention of Skp2 protein at early time points post serum release (similar to early G1 phase) to ensure sufficient ubiquitination of Skp2, leading to the elevated degradation of Skp2 protein at later timepoints (Fig. 4D). The degradation of Skp2 restored the protein level of p27^Kip1^ and induced the cell cycle arrest, proliferation and migration inhibition in STS cells (Fig. 6). Our findings unveil a new role of RCC1 in promoting the nucleo-cytoplasmic trafficking of oncogenic Skp2 protein, the process of which is commonly observed and correlated with the progression of many types of cancer [7, 8]. Hence, RCC1 might be a promising target for improving the treatment of STS in clinics.

RCC1 has been known as a regulator in the processes of the nucleo-cytoplasmic transport, DNA synthesis, nuclear membrane disintegration, mitotic spindle assembly and oncogenesis [20, 27, 29]. Interestingly, the expression and function of RCC1 vary in a context-dependent manner. RCC1 is negatively correlated with the progression of lung adenocarcinoma and gastric cancer [20]. On the other hand, RCC1 is found to be upregulated and plays oncogenic roles in many other types of cancer, such as cervical cancer [15] and colon cancer [13]. For example, in human papillomavirus E7-expressing cervical cancer, RCC1 is found to be upregulated and promoted tumorigenesis by abrogating the G1 cell cycle checkpoint [15]. Abundant evidence suggests that RCC1 might act as an intermediate regulator in the process of proteins nucleo-cytoplasmic trafficking by promoting the formation of trimeric “exportin 1 (XPO1)-cargo-RanGTP” complex [25]. Thus, the role of RCC1 in tumorigenesis might depend on the cargo proteins it associates with in specific cancer types. In our study, we showed that knockdown of RCC1 induces the nucleus retention of Skp2 protein, while overexpression of RCC1 promotes the cytoplasmic distribution of Skp2. Importantly, the elevated cytoplasmic Skp2 distribution is observed and associated with the progression of malignancy in several cancer types [30, 31]. Nonetheless, the regulatory mechanisms underlying the nucleo-cytoplasmic trafficking of Skp2 is not completely elucidated. The classical model of “XPO1 dependent nucleo-cytoplasmic shuttling” depends on the presence of nucleus exporting sequence (NES) motif, which is absent in Skp2 [26]. This discrepancy suggests that there must be some cofactor proteins involved in the nucleo-cytoplasmic trafficking of Skp2 in STS. Indeed, by protein co-IP assay, we revealed a direct binding between Skp2 and RCC1, either endogenously or exogenously (Fig. 4E, F). These results suggest that RCC1 might associate with Skp2 and facilitate the formation of exportation complex for Skp2.

The dysregulation of the Skp2-p27^Kip1^ axis, manifested as either Skp2 upregulation or p27^Kip1^ downregulation, is a common event in many types of human malignancies [32]. Especially, significant elevation of Skp2 abundance is commonly found in STS samples and correlated with a poor prognosis [33]. It’s worth noting that overexpression of Skp2 is required for the survival of aggressive cancer cells harboring multiple mutations of tumor suppressor genes [3, 19, 34]. Also, pharmacological inhibition of Skp2 has been shown to limit cancer stem cell traits and impede cancer progression [35–37]. Post-translational modifications of Skp2 have been reported to regulate its function by governing the subcellular location of Skp2. Phosphorylation of Ser72 by Akt and acetylation of K68 and K71 mediated by p300 contribute to the cytoplasmic localization and stability of Skp2, subsequently leading to the degradation of p27^Kip1^ and E-cadherin in breast cancer [8], prostate cancer [38], and liver cancer [39]. However, the detailed mechanism underlying the nucleus export process of Skp2 remains unclear [40]. Our results suggest that RCC1 might function as a cofactor to assist the nucleo-cytoplasmic shuttling of Skp2. Targeting RCC1 would disrupt this process, leading to the nucleus retention of Skp2 and a subsequent reduction in its cytoplasmic abundance. Further investigation regarding how post-translational modifications of Skp2 interact with the RCC1 mediated nucleus exportation process would be interesting.

In summary, our results contribute to the elucidation of the nucleus exportation of oncogenic Skp2 protein. The identified RCC1-Skp2-p27^Kip1^ axis might benefit the development of therapeutic strategies for STS. It’s worth noting that targeted inhibition of RCC1 was reported to restore sensitivity to immunotherapy and chemotherapy [13, 16]. This suggests that targeting RCC1 might have potential in combinatory therapy with first-line anti-cancer drugs like EGFR inhibitors [41], mTOR inhibitors [42] and AR inhibitors [43], whose effectiveness are often compromised by increased cytoplasmic distribution of Skp2.

### Supplementary information

Supplementary information

Original Data File

## Data Availability

Data for differential gene expression and survival analysis are publicly accessible. Data were sourced from GEPIA (http://gepia.cancer-pku.cn/index.html), TCGA-SARC cohort data from the GDC Data Portal (https://portal.gdc.cancer.gov/), and GES 30929 data from NCBI’s GEO. The remaining data are available in the article, Supplementary Information, or the Original Data file, and can also be obtained from the corresponding authors upon a reasonable request.
