# Supplementary material for: Targeting RCC1 to block the human soft-tissue sarcoma by disrupting nucleo-cytoplasmic trafficking of Skp2
Source: Cell Death Dis. 2024 Apr 1;15(4):241. doi: 10.1038/s41419-024-06629-2 (PMC10985091; doi:10.1038/s41419-024-06629-2)

Supplementary figures

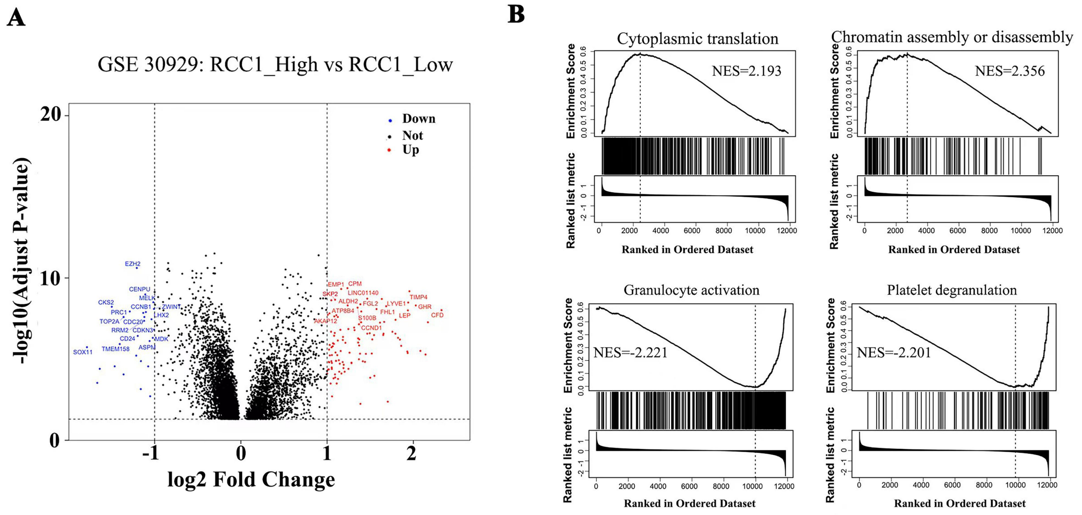

Figure S1 Different expression profiles of stromal genes as response to high expression of RCC1.

A Volcano plot of expression data. Significant differential expressed genes were marked with red or blue plots. **B** Gene set enrichment analysis (GSEA) showed that the samples with high RCC1 expression enriched in cytoplasmic translation (NES = 2.193), Chromatin assembly or disassembly (NES = 2.356). The most downregulated gene sets with high RCC1 expression were clustered in granulocyte activation (NES = -2.221), platelet degranulation (NES = -2.201).

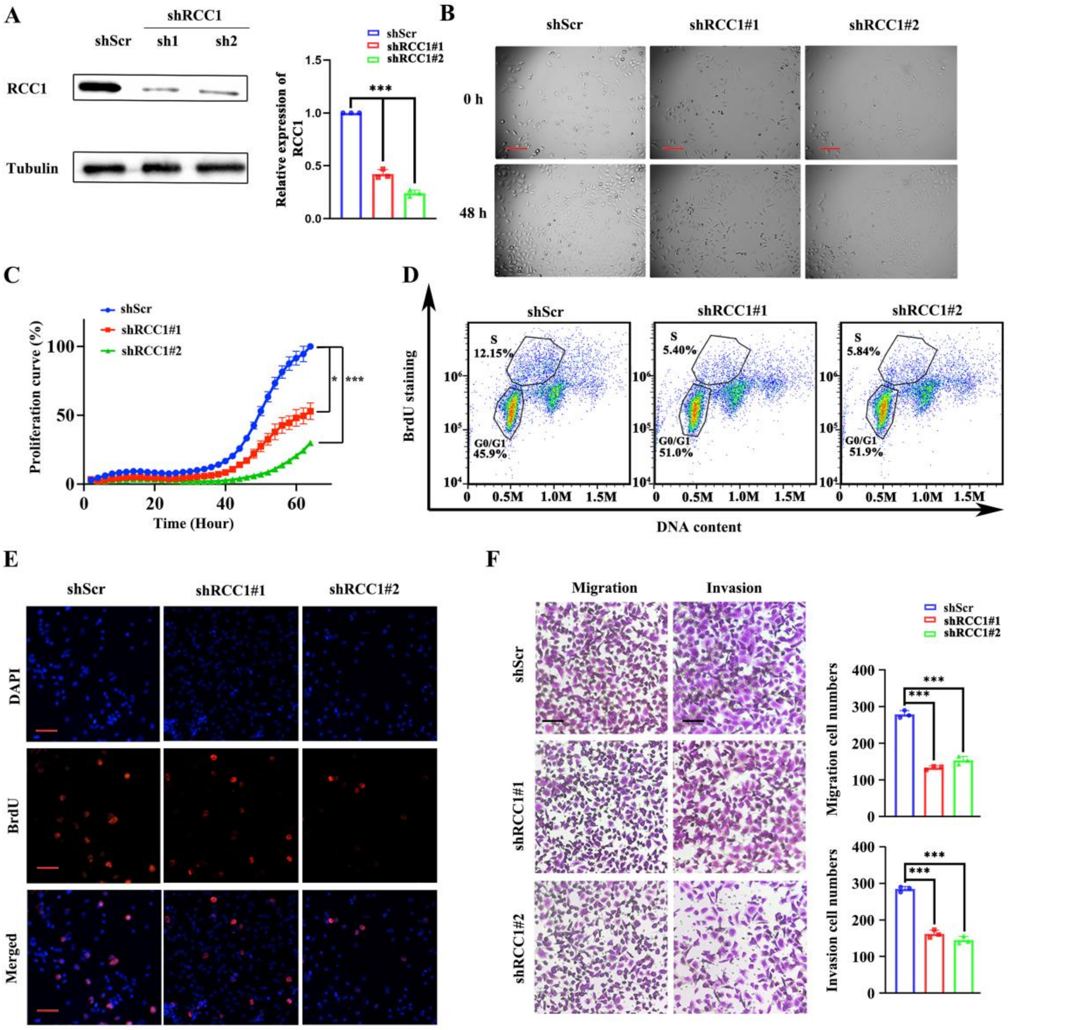

Figure S2 Knockdown of RCC1 suppressed the proliferation, cell cycle transition, migration and invasion of HTC75.

A Western blot analysis of RCC1 knockdown soft tissue sarcoma cell line HTC75. **B** Endpoint bright field images of RCC1 knockdown HTC75 cell lines by using live cell imaging system ZenCell owl. **C** Real-time cellular analysis of RCC1 knockdown HTC75 cell lines. **D** FACS analyses using BrdU and PI staining for shScr and RCC1-KD (shRCC1#1, shRCC1#2) HTC75 cells. The cells belong to different cell cycle sub-phase is determined within the circle, with the percentage shown close to it. **E** Immunofluorescence images of indicated cells stained by pulse-incorporation of BrdU and immunostaining using anti-BrdU antibody (red). DAPI was used to counterstain the cells nuclei (blue). Scale bars: 50 μm. **F** Knockdown of RCC1 decreased migration and invasion of HTC75 cells. The cells in five randomly selected fields were counted and statistically analyzed. The number of migration/invasion cells per field was fewer in shRCC1 cells compared with shScr cells. Significance (*$p < 0.05$, ***$p < 0.001$). Scale bars: 100 μm.

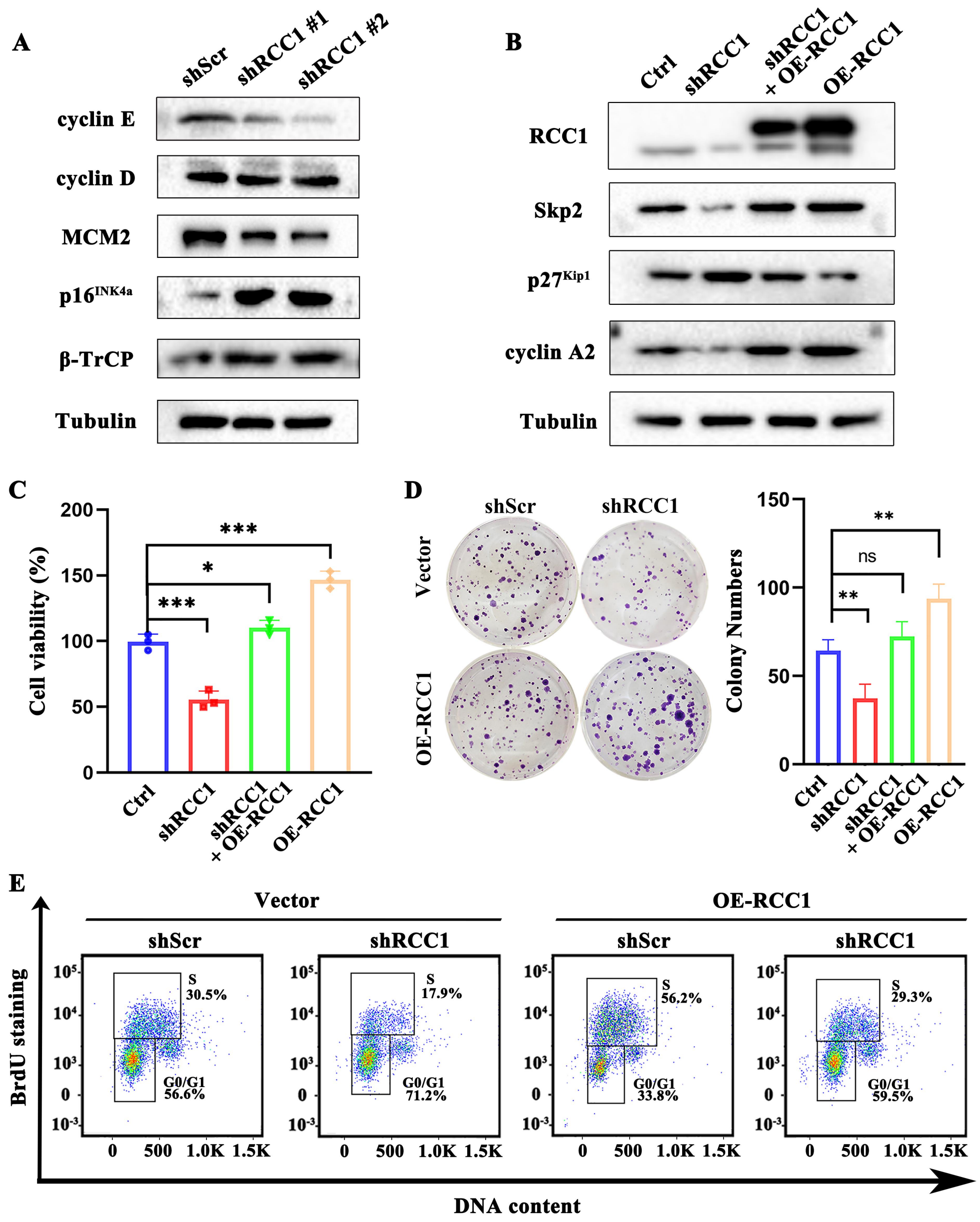

Figure S3 Exogenous expression of RCC1 promoted the accumulation of Skp2 and G1/S phase transition.

A Immunoblotting analysis of cyclin D, cyclin E, MCM2, p16^{Ink4}, p57 and β-TrCP in RCC1 knockdown SW872 cells. Tubulin was used as the loading control. **B** Immunoblotting analysis demonstrates the expression levels of Skp2, p27^{Kip1}, and cyclin A2 in response to RCC1 knockdown (shRCC1), RCC1 knockdown followed by exogenous RCC1 overexpression (shRCC1+OE-RCC1) in the same cells, and RCC1 overexpression in SW872 cell lines. **C & D** Cell viability and cell colony formation abilityof SW872. **E** FACS analyses of control, RCC1 knockdown or overexpression cells. The cells belonging to different cell cycle sub-phase are determined, with the percentage shown alongside using barchart. Data is expressed as mean ± SD (n = 3). Significance (*$p < 0.05$, **$p < 0.01$, ***$p < 0.001$).

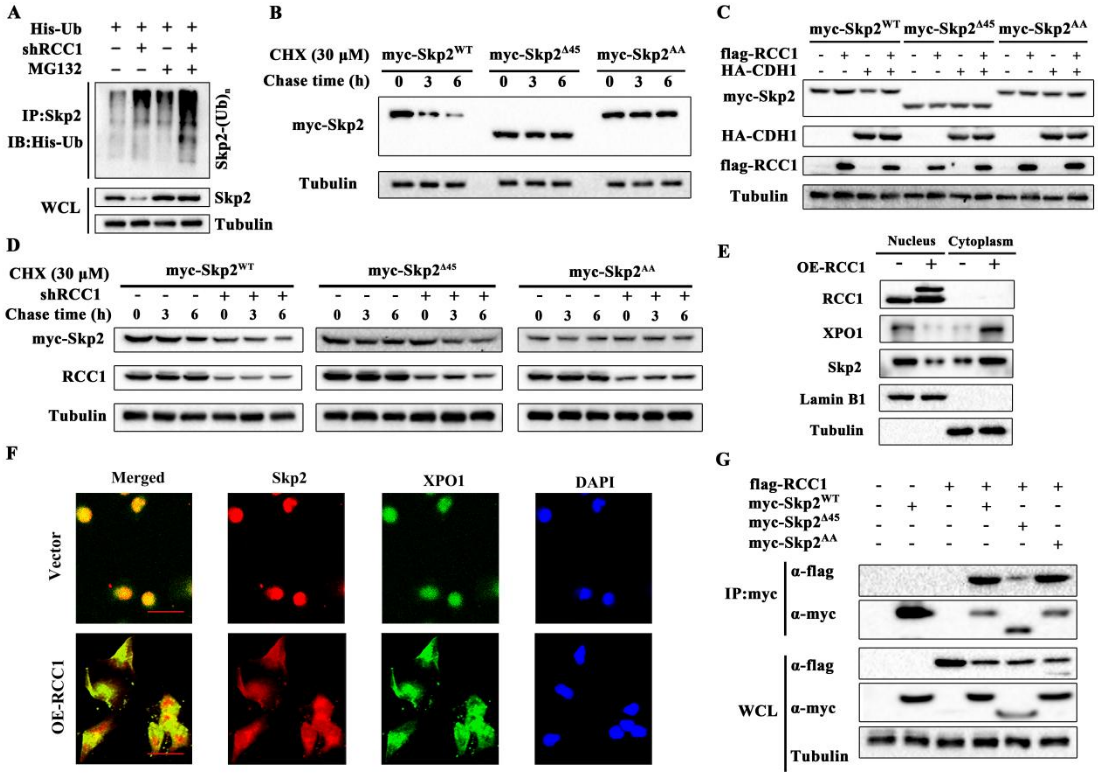

Figure S4 Exogenous expression of RCC1 protect Skp2 from CDH1-mediated degradation by binding to the N-terminal of Skp2.

A *In vivo* endogenous ubiquitination assay to detect ubiquitination of Skp2 influenced by RCC1 knockdown on SW872 cell. **B** Cycloheximide (CHX) assays showed that the degradation rate of Skp2 D-box mutants (Skp2$^{\Delta45}$, Skp2^{AA}) is slower than wild-type Skp2. Indicated cells were treated with cycloheximide (CHX, 30 μM) to inhibit protein synthesis, and harvested at indicated time-points for immunoblotting analysis. Tubulin was used as the loading control. **C** The differential protection effect of RCC1 on CDH1-midiated degradation of Skp2^{WT}, Skp2$^{\Delta45}$ and Skp2^{AA}. **D** Cycloheximide (CHX) assays were conducted to investigate whether knockdown of RCC1 promoted the degradation rate of Skp2^{WT}, Skp2$^{\Delta45}$ and Skp2^{AA}. **E** Immunoblotting analysis of subcellular localization of Xpo1 and Skp2 in RCC1 overexpression SW872 cells. **F** Immunofluorescence analysis was performed to determine the subcellular localization of XPO1 (green) and Skp2 (red) in SW872 cells with RCC1 overexpression. DAPI was used to counterstain the cells nuclei (blue). Scale bars: 50 μm. **G** *In vivo* protein co-immunoprecipitation of Skp2 and Skp2 D-box mutants (Skp2$^{\Delta45}$, Skp2^{AA}) with RCC1 in HKE293t cells.

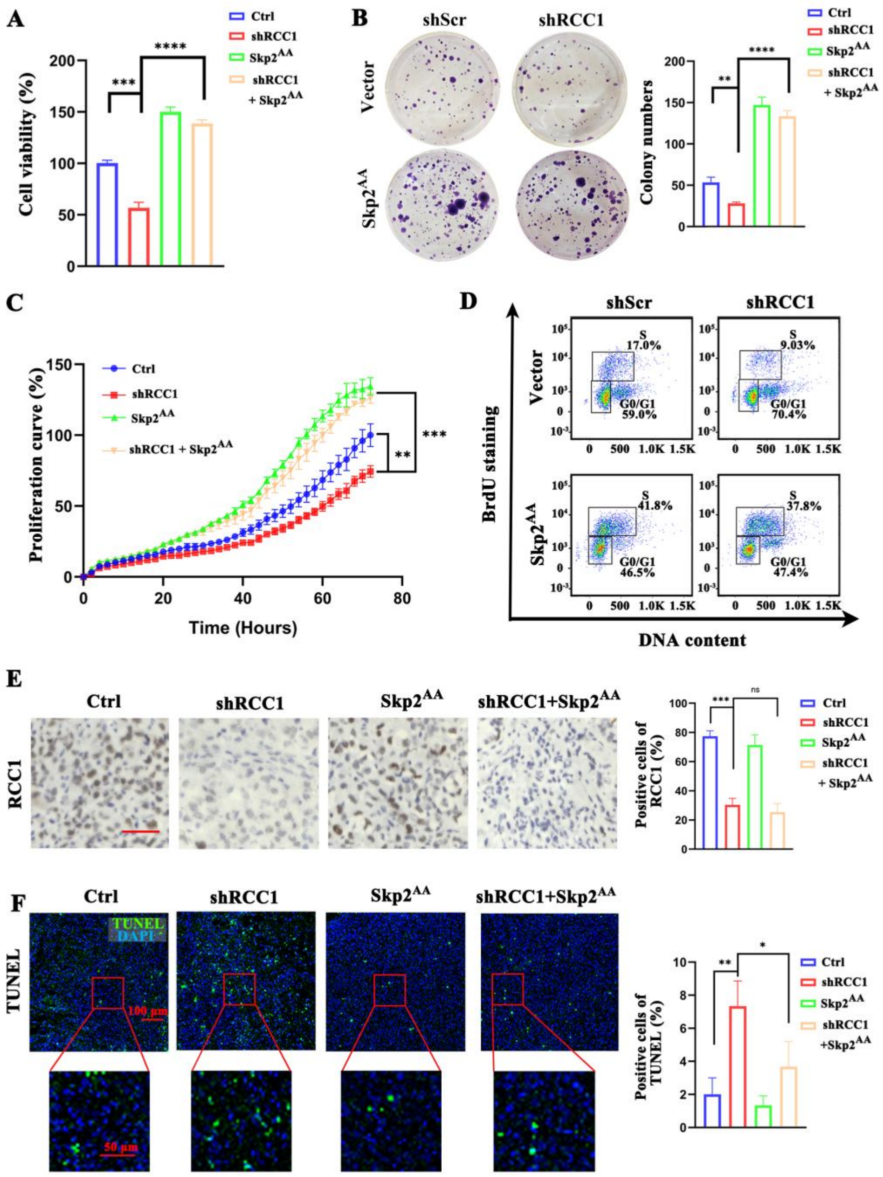

Figure S5 Expression of non-degradable Skp2 resume tumor suppression triggered by RCC1 knockdown.

A Cell viability of shScr, shRCC1, Skp2^{AA} and shRCC1+Skp2^{AA} cells, respectively. **B** Colony formation assay of shScr, shRCC1, Skp2^{AA} and shRCC1+Skp2^{AA} cells. Bar graph quantifications representing the colony numbers are presented alongside. **C** Real-time cellular analysis (RTCA) of shScr, shRCC1, Skp2^{AA} and shRCC1+Skp2^{AA} cells. **D** FACS analyses using BrdU and PI staining for shScr, shRCC1, Skp2^{AA} and shRCC1+Skp2^{AA} cells. **E** Immunohistochemistry (IHC) of RCC1 of xenograft tumor sections. Percentage of IHC positive cells is quantified alongside. Scale bars: 50 μm. **F** TUNEL apoptosis assay for xenograft tumor sections from indicated groups were presented. Scale bars : 100 μm (low magnification) and 50 μm (high magnification). Data are presented as mean ± SD (n = 3, $^{**}p < 0.01$, $^{***}p < 0.001$).

Supplementary table

Supplementary Table 1: qPCR Primer list

**RT-qPCR primers**	
1.RCC1 （h）	
sense	GCCTCAGGAAACGACCACTT
antisense	CCACCACGGTTGGCAAATAA
2.SKP2(h）	
sense	AGGAGCCCGACAGTGAGAA
antisense	GGGAGGCACAGACAGGAAA
3. *GAPDH* (h)	
sense	AACGGATTTGGTCGTATTGGG
antisense	CCTGGAAGATGGTGATGGGAT
4.MCM3(h)	
sense	TCTGCGGTATGTGCTTTGC
antisense	AAACCACGCCTCGGTCAGC
5.E2F1(h)	
sense	AAGAACCGCTGTTGTCCCG
antisense	GAGGCCGAAGTGGTAGTCG
6. PCNA(h)	
sense	TCAAGAAGGTGTTGGAGGCA
antisense	TGGGACGAGTCCATGCTCTG
7.CDKN1B(p27,h)	
sense	CGGCTAACTCTGAGGACACG
antisense	TTCTGAGGCCAGGCTTCTT
8.CCNE(h)	
sense	ATGTCACCGTTCCTCCTTGG
antisense	GGGCATCTTCACGCTCTAT
antisense	AGAAGGTCCGAGCACA
9.MCM4(h)	
sense	TGTTTTTCCA GCCCTCCCCAAATG
antisense	GAG TGCCGTATGTCAGTGGTGAAC
10.MCM5(h)	
sense	GTTTGACAAGATGCGAGAA
antisense	CCTTGGCGATAGAGATGG
11. MCM6(h)	
sense	GTGATCAGGGATGTAGAACAGC
antisense	AGCTTGGGTCTCTTGAATACG
12.MCM7(h)	
sense	ACCGAGACAATGACCTAC

antisense	GCTATGTAACGCCTCATG
13.p16^{INK4a}(h)	
sense	CAACGCACCGAATAGTTACG
antisense	ACCACCAGCGTGTCCAGGAA
14.p57(h)	
sense	AACGCCGAGGACCAGAACC
antisense	GCGAAGAAATCTGCACCGTCT
15. cyclin D(h)	
sense	TTCTTGAGCAACACCCTCTTCTGCAGCC
antisense	TCGCCATATACCGGTCAAAGAAATCTTGTGCC
16. *GAPDH* (h)	
sense	AACGGATTTGGTCGTATTGGG
antisense	CCTGGAAGATGGTGATGGGAT

Supplementary Table 2 GSEA Enrichment Results

Gene Set	Description	Size	Leading Edge Number	ES	NES	p-Value
GO:0006260	DNA replication	211	103	0.63421	2.6472	<2.2e-16
GO:0006397	mRNA processing	365	191	0.58247	2.5715	<2.2e-16
GO:0006333	chromatin assembly or disassembly	119	64	0.60936	2.3562	<2.2e-16
GO:0006302	double-strand break repair	151	73	0.57265	2.2768	<2.2e-16
GO:0006338	chromatin remodeling	120	52	0.59052	2.2756	<2.2e-16
GO:0000075	cell cycle checkpoint	177	78	0.54679	2.2678	<2.2e-16
GO:0002181	cytoplasmic translation	69	42	0.62451	2.1982	<2.2e-16
GO:0006310	DNA recombination	199	78	0.52725	2.1867	<2.2e-16
GO:0006284	base-excision repair	40	26	0.66656	2.1734	<2.2e-16
GO:0006353	DNA-templated transcription, termination	59	40	0.62068	2.1411	<2.2e-16
GO:0016999	antibiotic metabolic process	113	34	-0.6451	-2.1144	<2.2e-16
GO:0006631	fatty acid metabolic process	273	88	-0.5936	-2.1201	<2.2e-16
GO:0006909	phagocytosis	203	93	-0.62772	-2.1692	<2.2e-16
GO:0002526	acute inflammatory response	131	49	-0.67186	-2.1956	<2.2e-16
GO:0002576	platelet degranulation	113	51	-0.68003	-2.2006	<2.2e-16
GO:0002446	neutrophil mediated immunity	417	161	-0.60767	-2.2137	<2.2e-16
GO:0036230	granulocyte activation	419	163	-0.609	-2.2143	<2.2e-16
GO:0016042	lipid catabolic process	234	73	-0.64038	-2.2183	<2.2e-16
GO:0006959	humoral immune response	201	60	-0.64697	-2.2344	<2.2e-16
GO:0050727	regulation of inflammatory response	289	100	-0.63489	-2.2421	<2.2e-16

Raw images related to Figure 4B

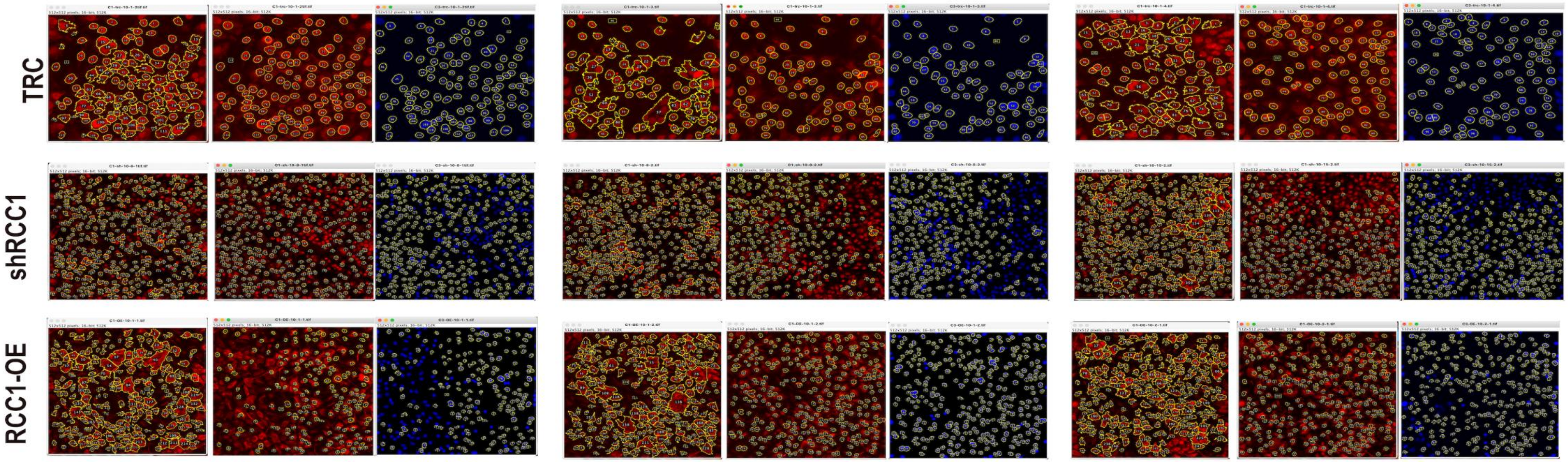

Supplement: Supplementary file 1 — Supplementary information [file 41419_2024_6629_MOESM1_ESM.pdf]
