# Supplementary material for: Targeting RCC1 to block the human soft-tissue sarcoma by disrupting nucleo-cytoplasmic trafficking of Skp2
Source: Cell Death Dis. 2024 Apr 1;15(4):241. doi: 10.1038/s41419-024-06629-2 (PMC10985091; doi:10.1038/s41419-024-06629-2)
Supplement: Supplementary file 2 — Original Data File [file 41419_2024_6629_MOESM2_ESM.pdf]

# Figure 2A

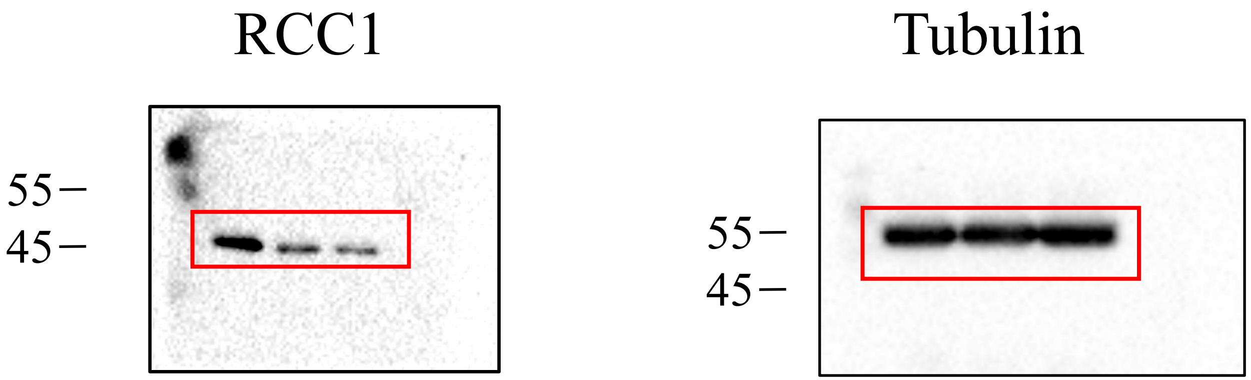


Uncropped raw images of western blotting and overlayed images with membranes shown in Figure 2A

Figure 3B

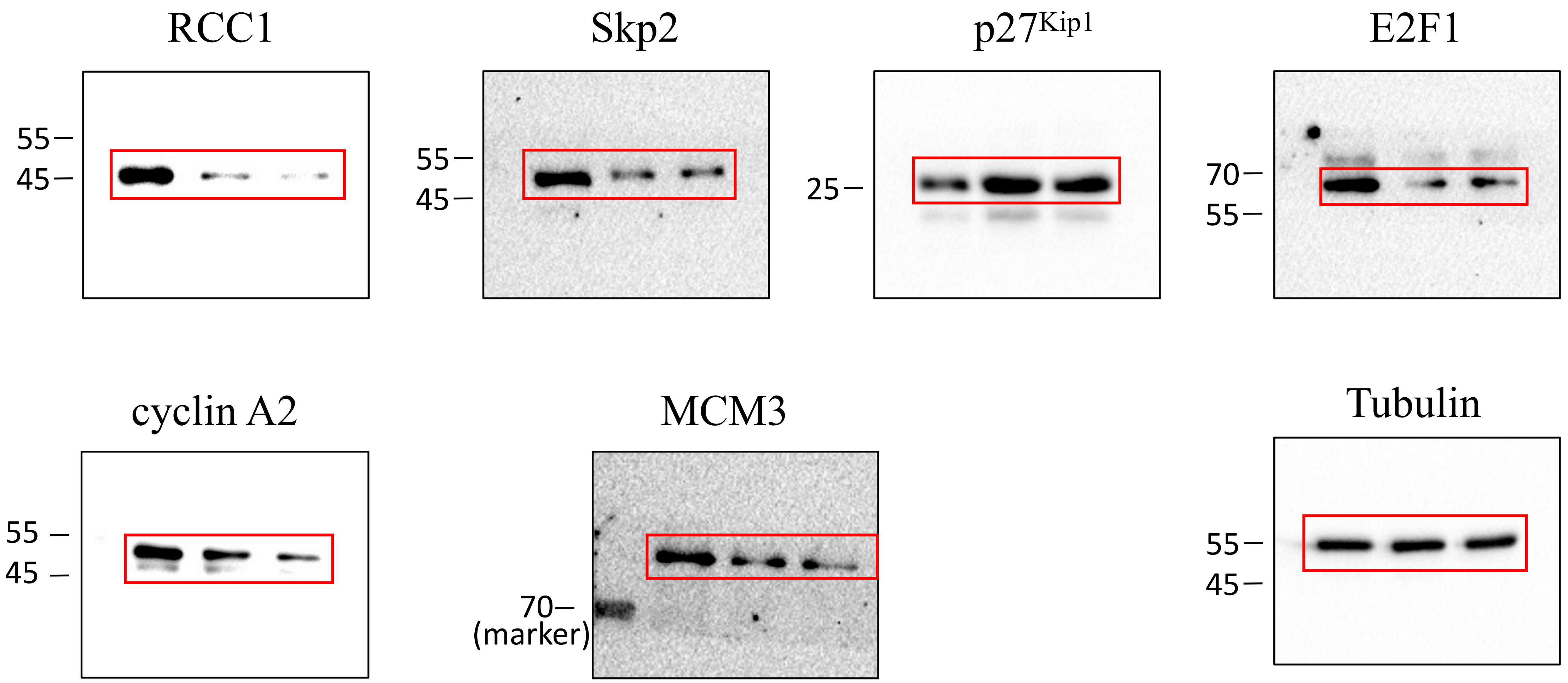


Uncropped raw images of western blotting and overlayed images with membranes shown in Figure 3B

Figure 3C

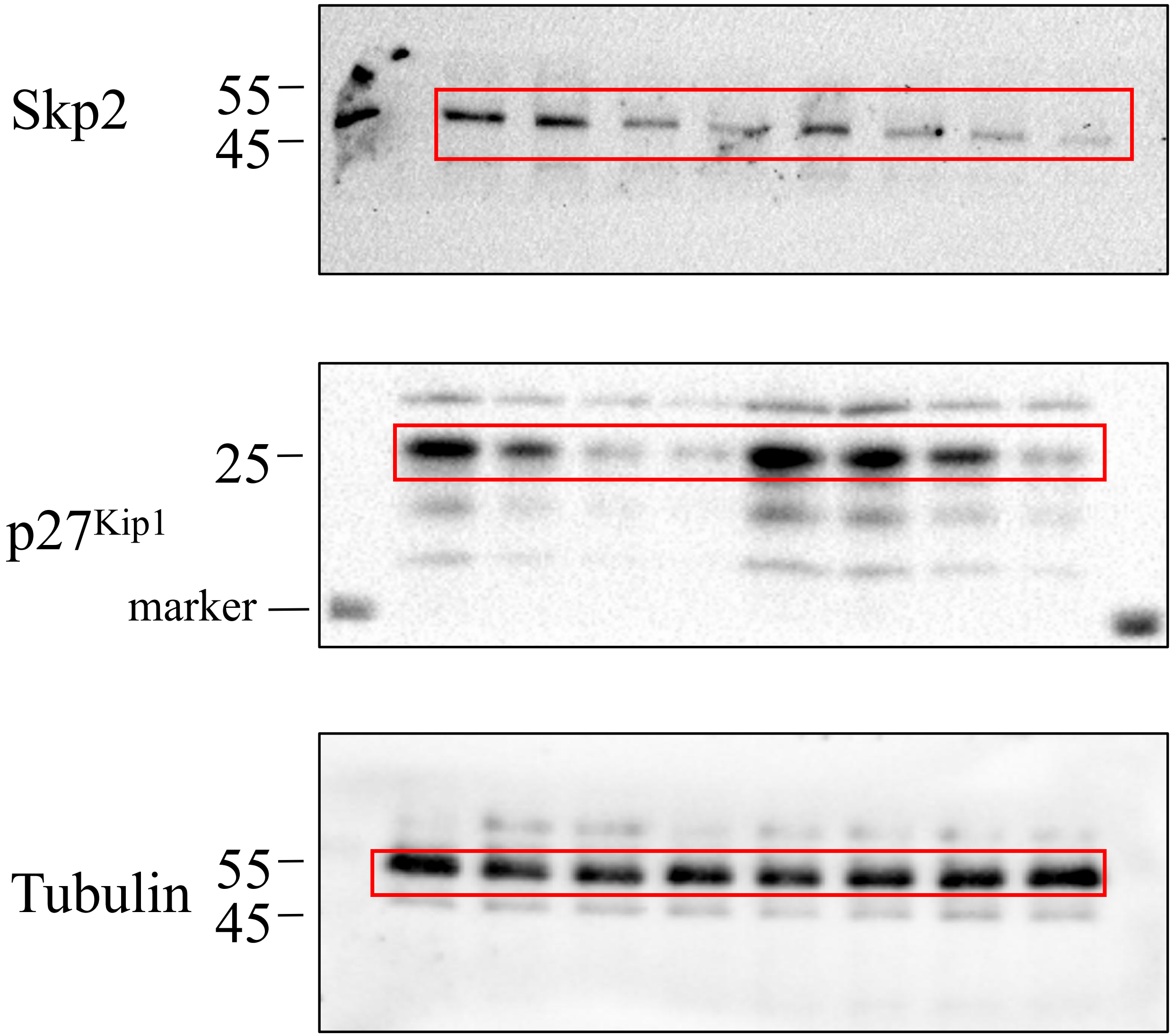


Figure 3D

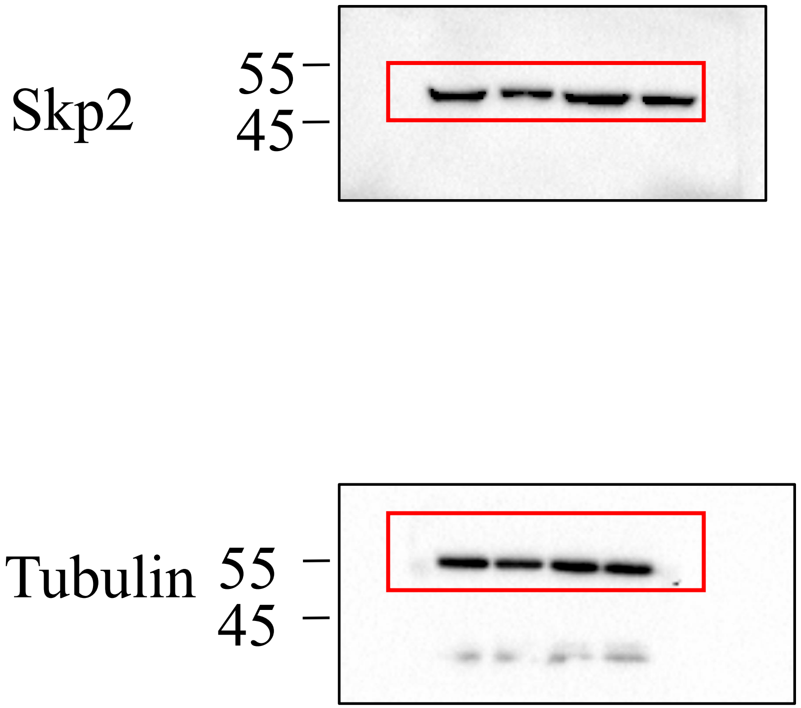


Uncropped raw images of western blotting and overlayed images with membranes shown in Figure 3C、3D

# Figure 4A

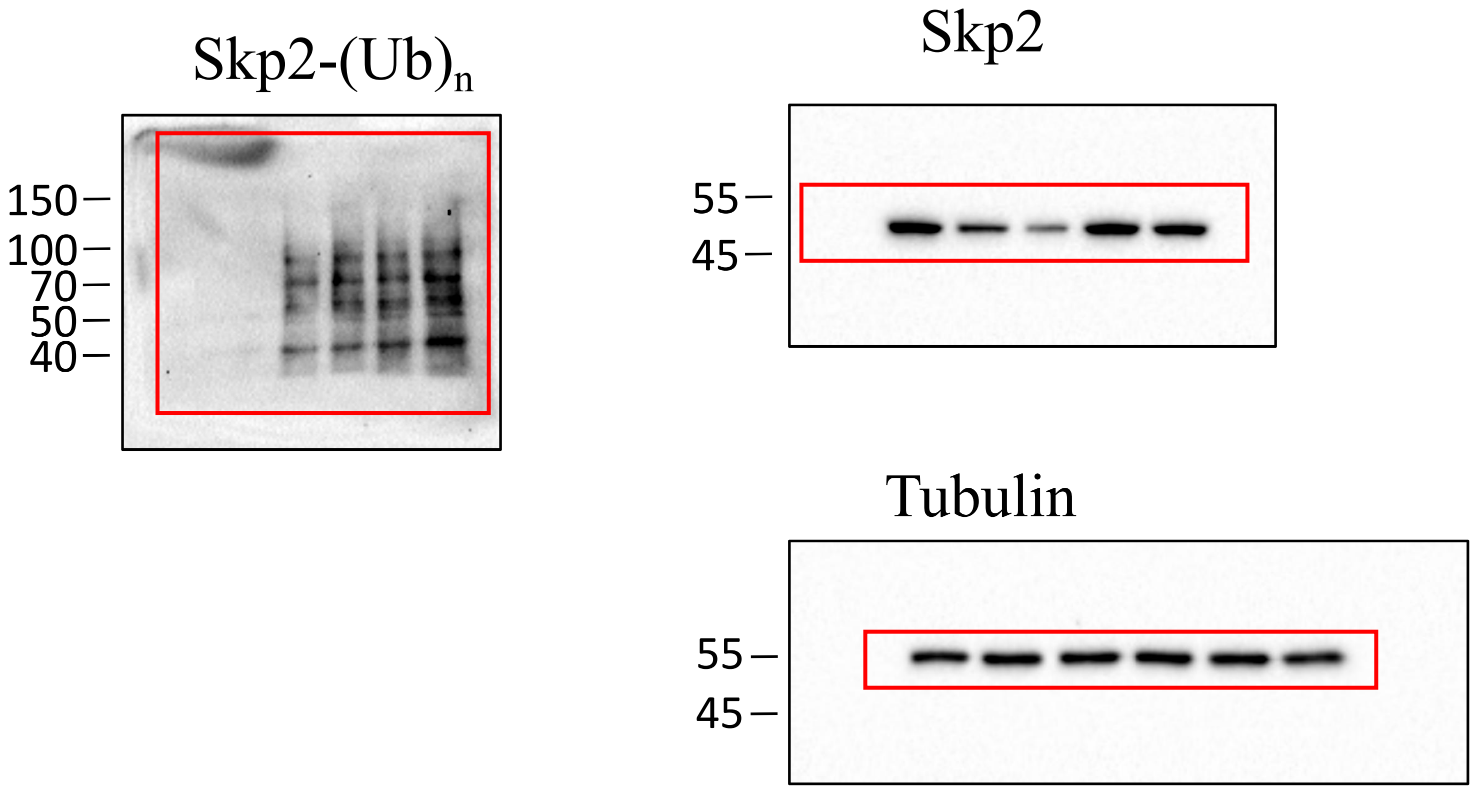


Uncropped raw images of western blotting and overlayed images with membranes shown in Figure 4A

Figure 4C

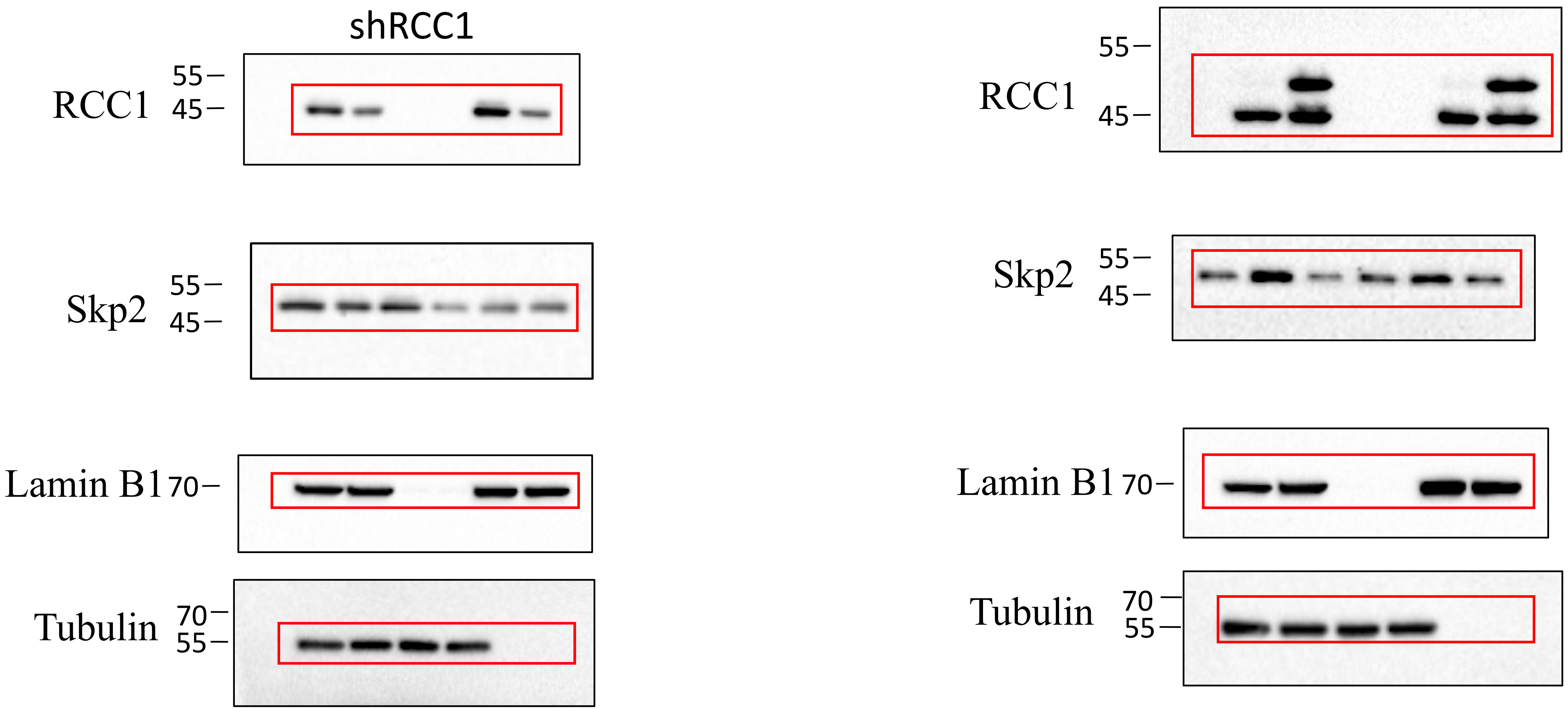


Uncropped raw images of western blotting and overlayed images with membranes shown in Figure4C

Figure 4D

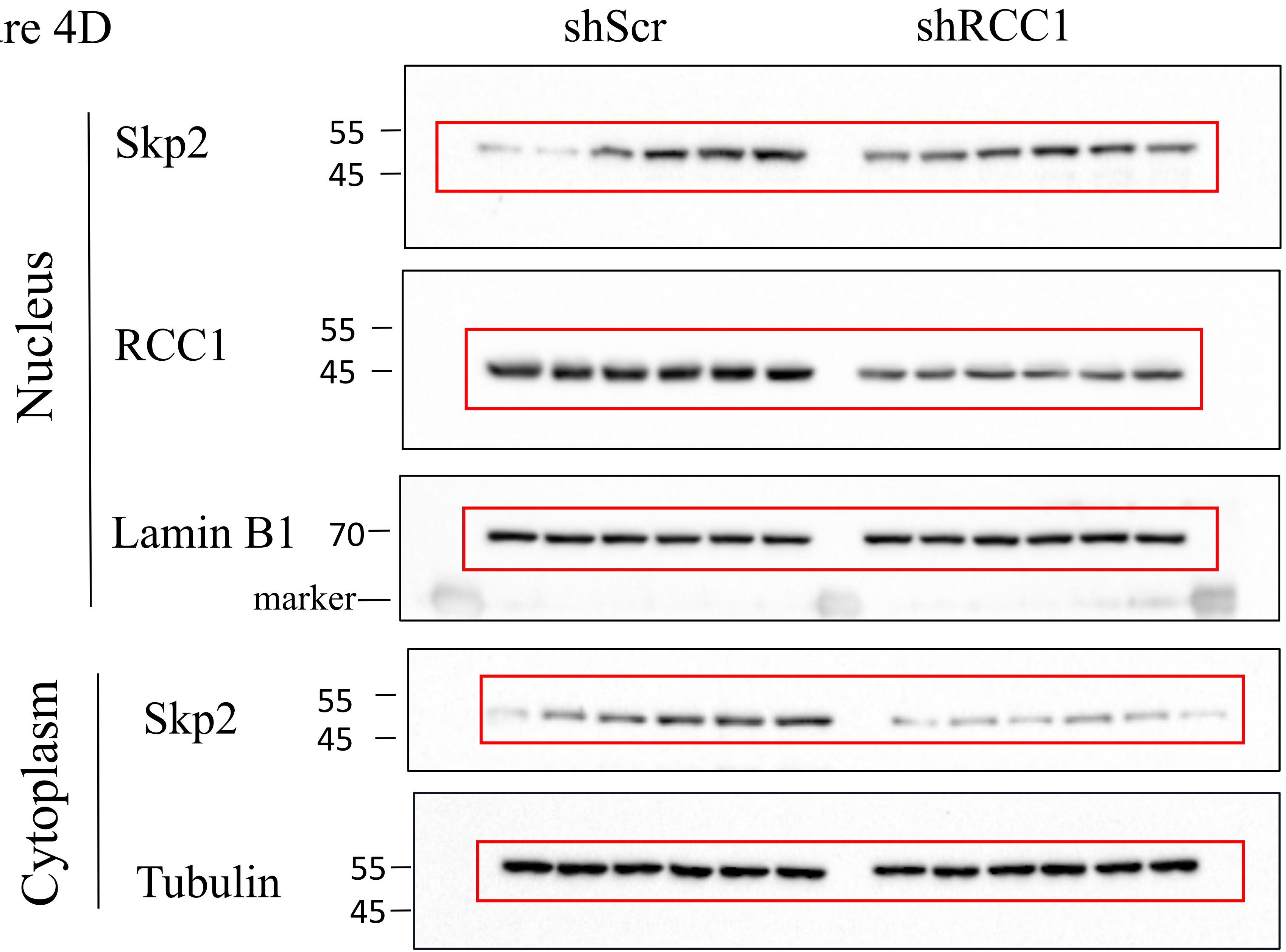


Uncropped raw images of western blotting and overlayed images with membranes shown in Figure 4D

Figure 4E

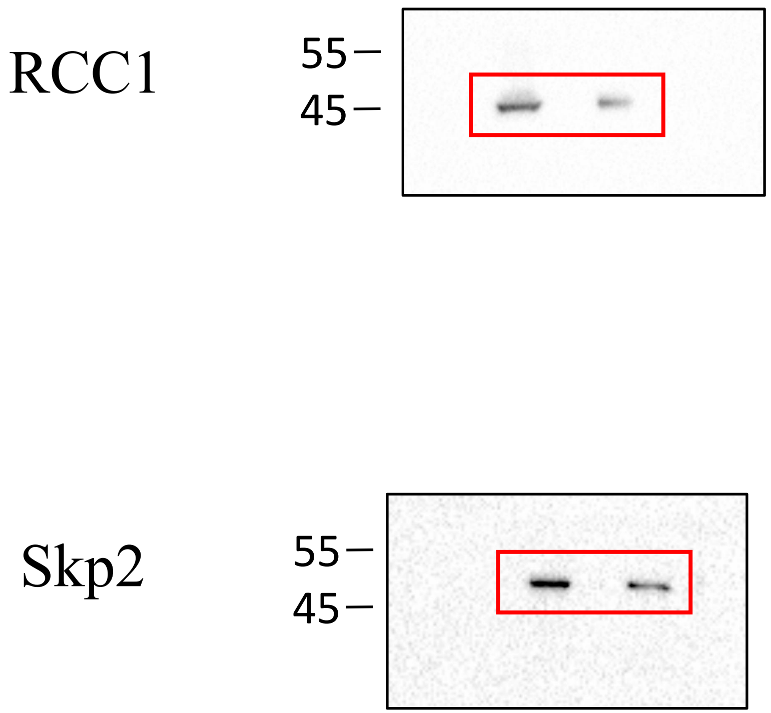


Figure 4F

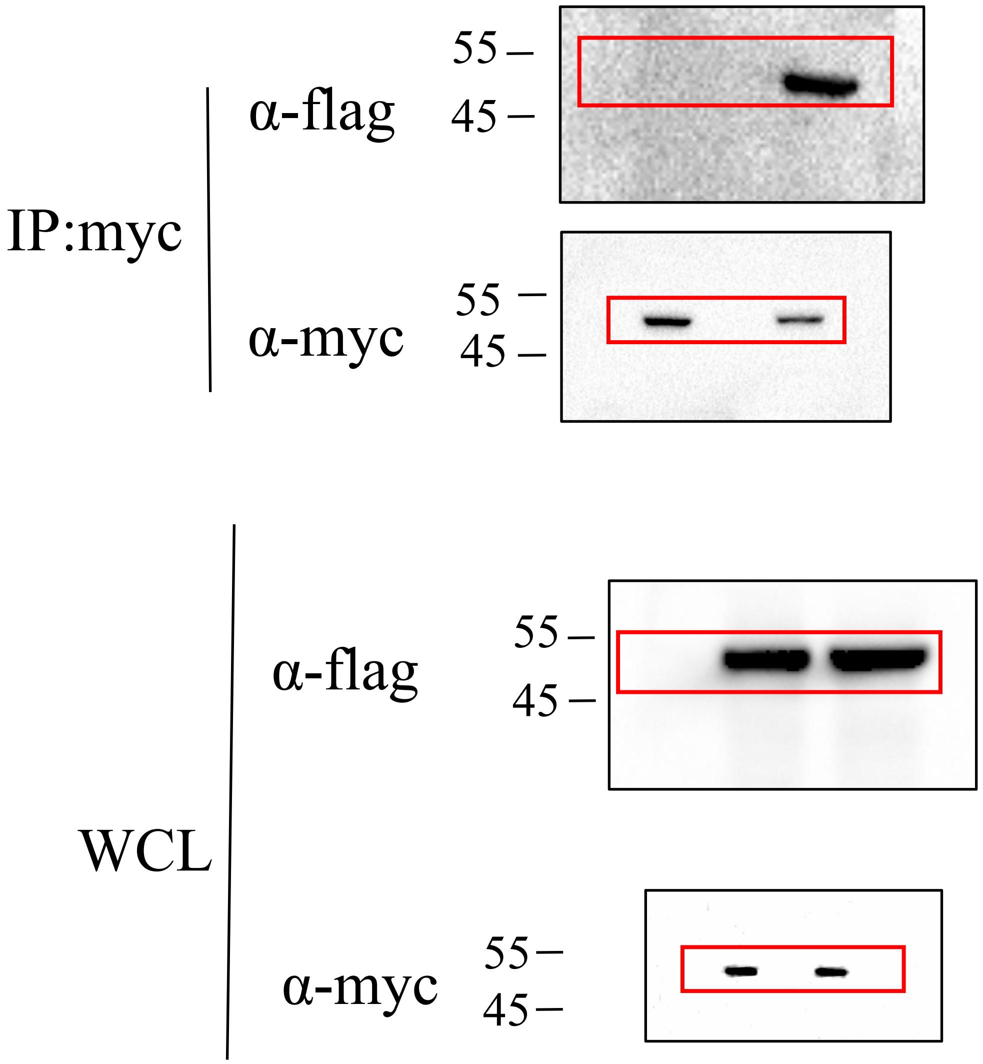


Uncropped raw images of western blotting and overlayed images with membranes shown in Figure 4E、4F

Figure 5E

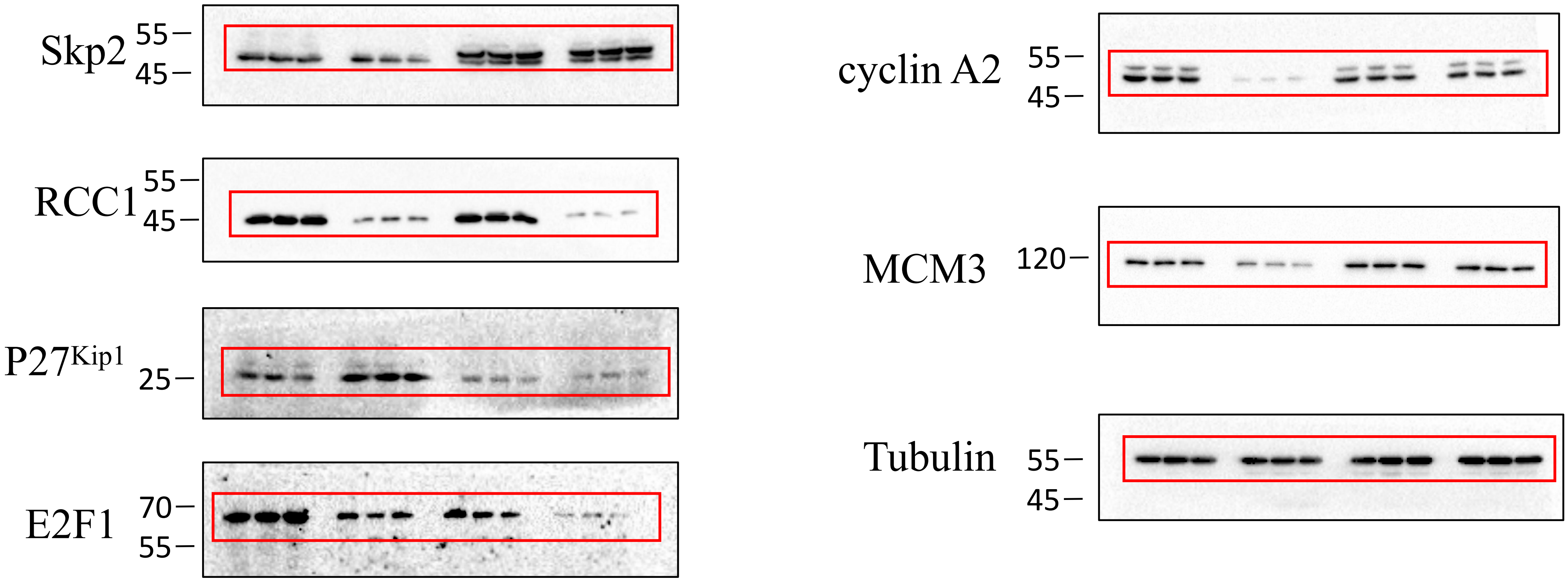


Uncropped raw images of western blotting and overlayed images with membranes shown in Figure 5E

# Figure S2

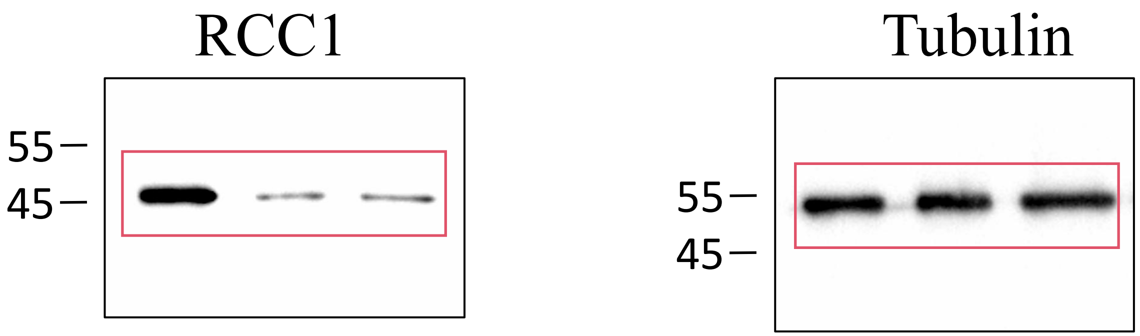


Uncropped raw images of western blotting and overlayed images with membranes shown in Figure S2

Figure S3A

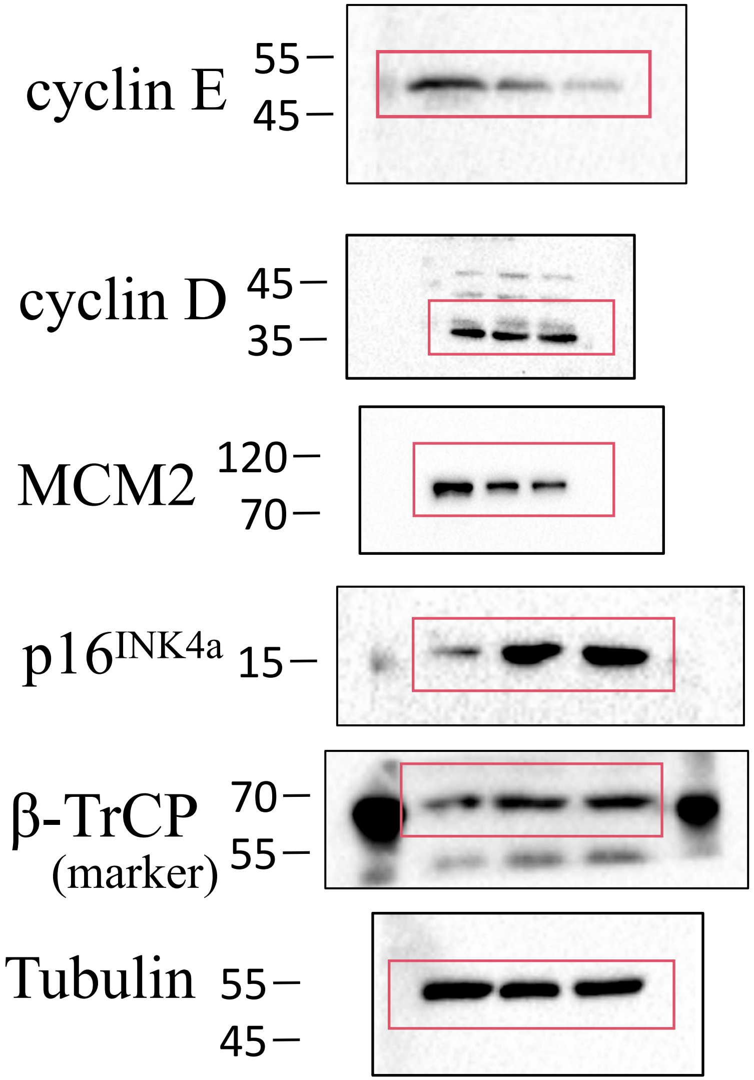


Figure S3B

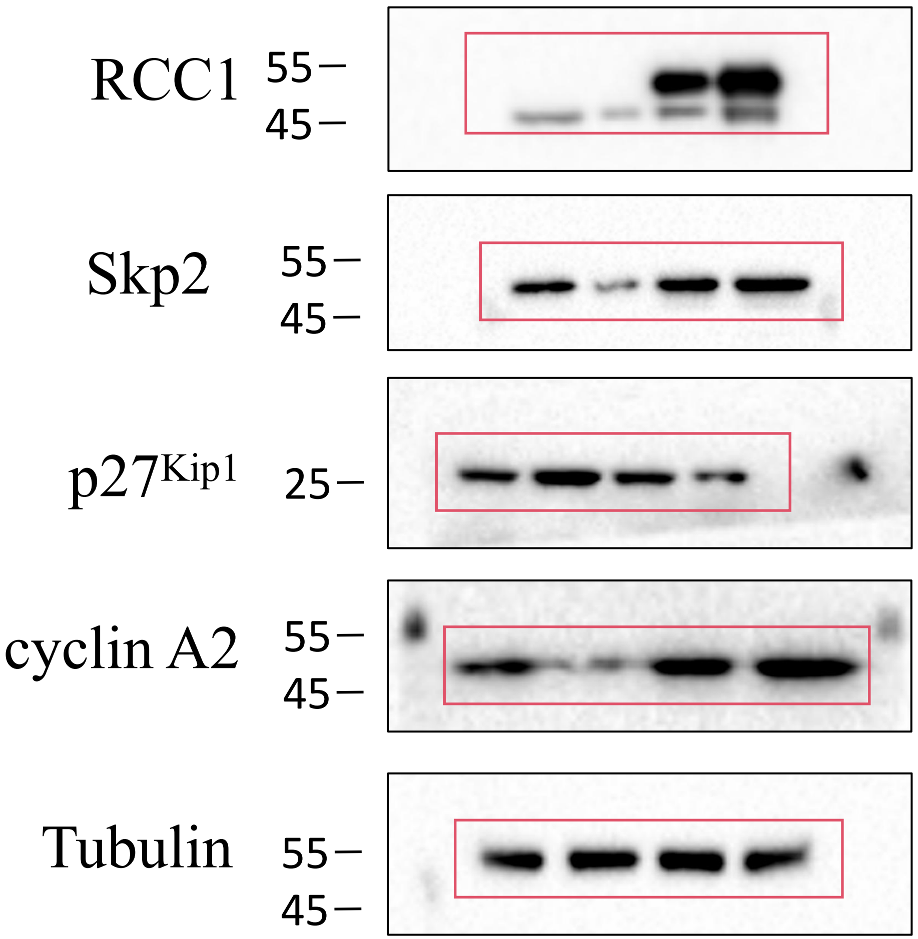


Uncropped raw images of western blotting and overlayed images with membranes shown in Figure S3A、S3B

Figure S4A

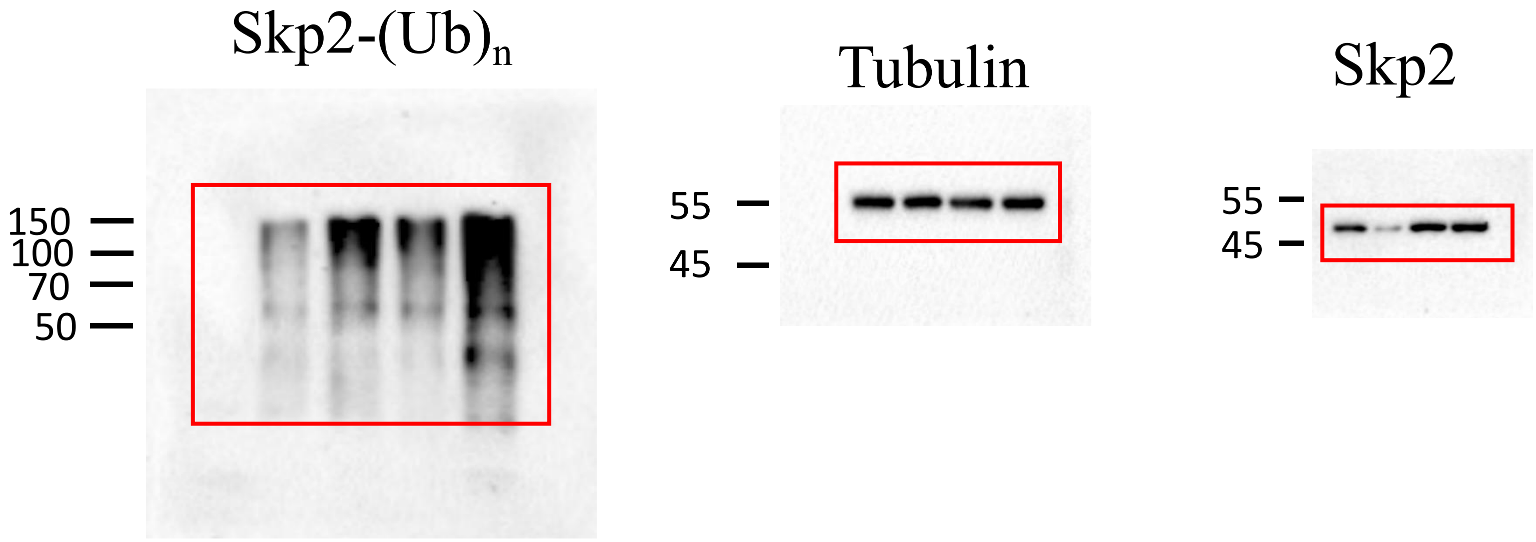


Uncropped raw images of western blotting and overlayed images with membranes shown in Figure S4A

Figure S4B

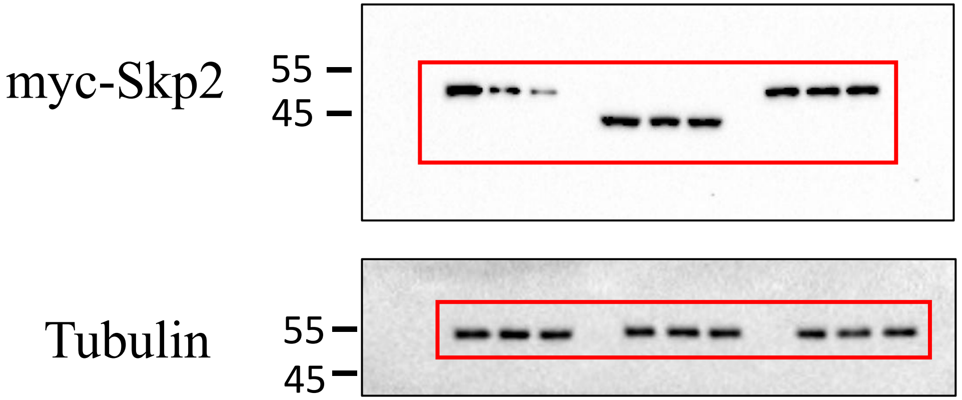


Figure S4C

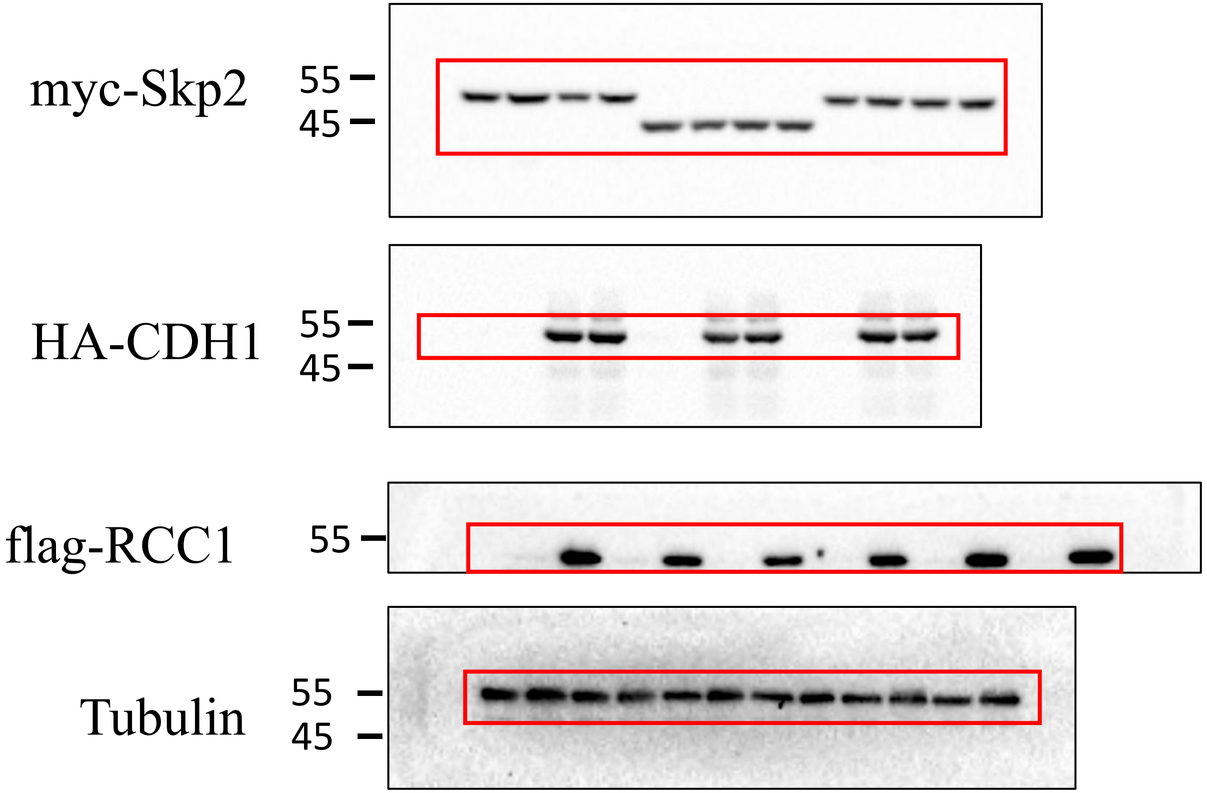


Uncropped raw images of western blotting and overlayed images with membranes shown in Figure S4B、4C

Figure S4D

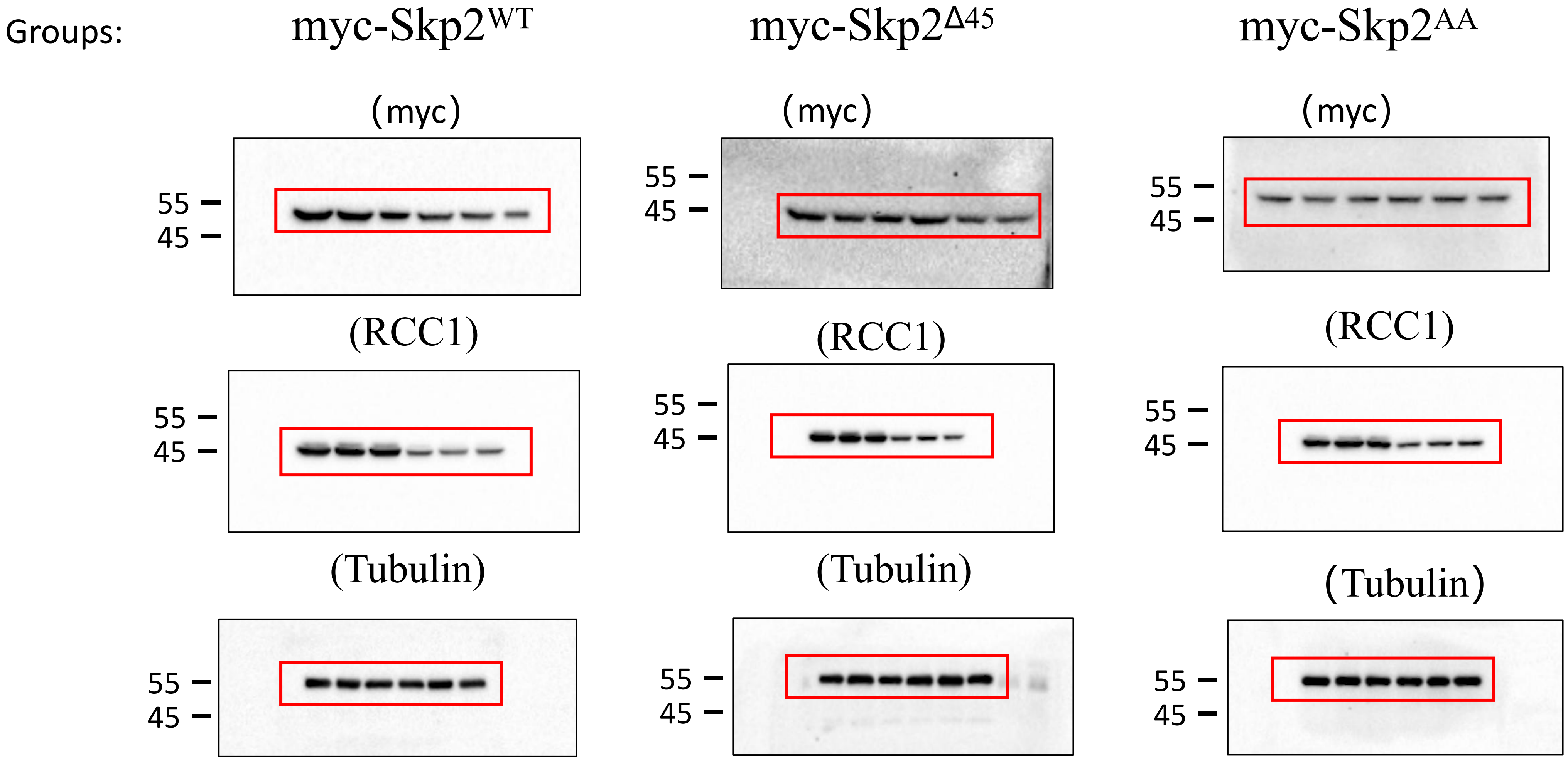


Uncropped raw images of western blotting and overlayed images with membranes shown in Figure S4D

# Figure S4E

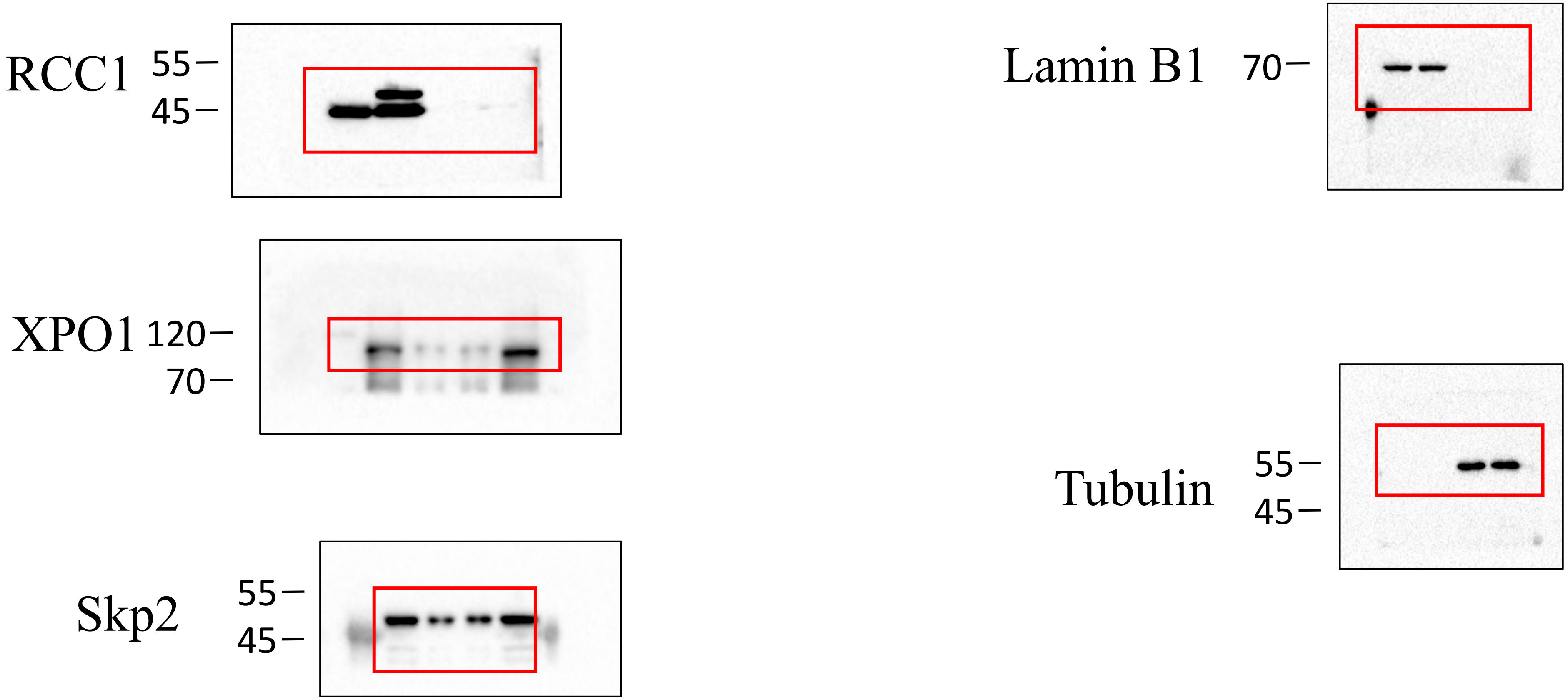


Uncropped raw images of western blotting and overlayed images with membranes shown in Figure S4E

Figure S4G

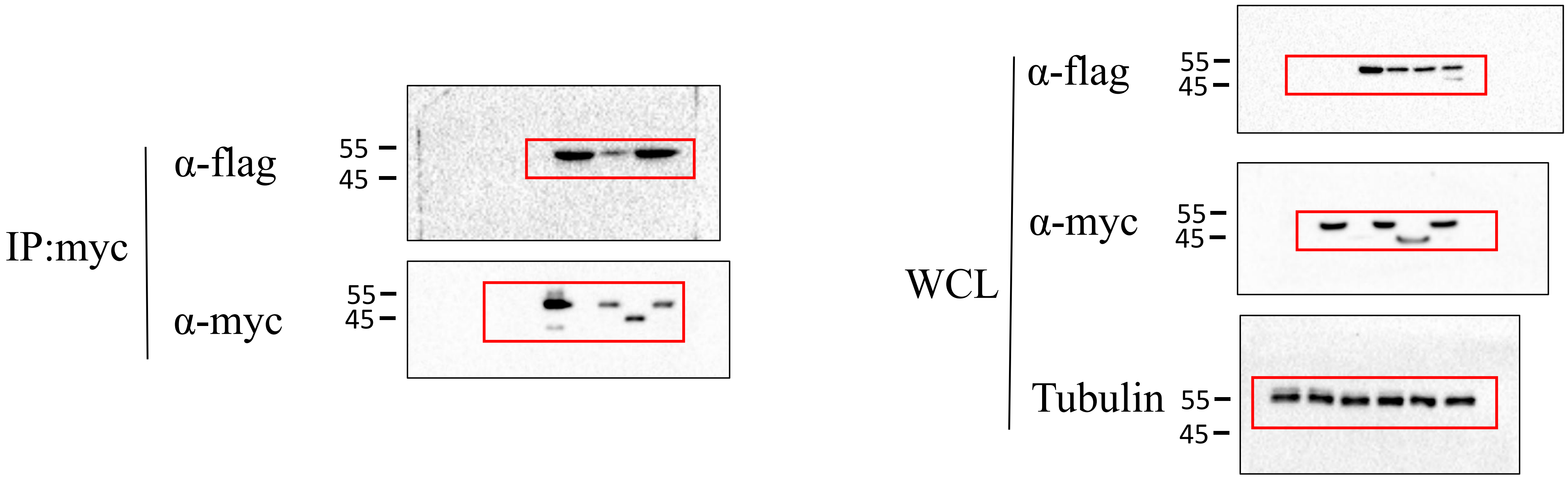


Uncropped raw images of western blotting and overlayed images with membranes shown in Figure S4G
